# Integrated biocontrol strategies using indigenous fungal endophytes *Aspergillus fumigatus* and *Curvularia lunata* against wheat stripe rust

**DOI:** 10.3389/fmicb.2025.1683295

**Published:** 2025-12-19

**Authors:** Salman Khan, Khalil Ur Rahman, Humaira Gul, Mamoona Rauf, Tsanko Gechev, Muhammad Arif, Akhtar Ali, Sajid Ali

**Affiliations:** 1 Department of Biotechnology Abdul Wali Khan University, Mardan, Pakistan; 2 Department of Botany Abdul Wali Khan University, Mardan, Pakistan; 3 Center of Plant Systems Biology and Biotechnology, Plovdiv, Bulgaria; 4 Department of Molecular Biology, Plovdiv University, Plovdiv, Bulgaria; 5 Global Plant Stress Research Center, Konkuk University, Seoul, Republic of Korea; 6 Department of Horticulture and Life Science, Yeungnam University, Daegu, Republic of Korea

**Keywords:** *Triticum aestivum* L., stripe rust, *Curvularia lunata*, *Aspergillus fumigatus*, endophytic fungi, biocontrol

## Abstract

**Background:**

Wheat stripe rust, caused by *Puccinia striiformis* f. sp. tritici (Pst), is a major pathogenic threat, particularly in regions with favorable moist conditions during the growing season, resulting in significant commercial losses. This study investigates the variations in wheat plant responses to pathogen stress and the potential biocontrol effects of fungal endophytes against stripe rust. Due to the challenges associated with culturing the obligate biotrophic basidiomycete fungi on artificial media, there is a dire need for eco-friendly, economical, and safe biocontrol alternatives.

**Methods:**

We explored the biocontrol potential of two indigenous fungal endophytes, *Curvularia lunata* (DT-4) and *Aspergillus fumigatus* (DT-8), against wheat stripe rust in two susceptible wheat varieties.

**Results:**

Our results revealed that both fungal strains significantly improved wheat grain germination and secondary metabolites induction in two wheat varieties. The Morocco variety showed enhanced seed germination (63.6 % DT-4, 72.7% DT-8), plant growth (48.9% DT-4, 55.6% DT-8), and seedling fresh weight (126% DT-4, 110% DT-8), highlighting their potential as biocontrol agents. Treated wheat plants with DT-4 and DT-8 consortia after infection with strip rust (*Puccinia striiformis*) suspension (SR-S) exhibited enhanced resistance to stripe rust, evidenced by increased antioxidant enzyme activities SOD, CAT, and POD (54.5, 54.6, 112.7%), reduced lipid peroxidation (42.1%), and decreased disease severity (80%). Similarly, wheat grain of TD-1 variety treated with fungus culture filtrate showed maximum germination for seeds (38.5% DT-4, 53.8% DT-8), plant growth (54.5% DT-4, 31.8% DT-8), and seedling fresh weight (125% DT-4, DT-8). A significant increase is observed in the antioxidant enzyme activities SOD, CAT, and POD (59.2, 71.9, 104.6%), reduction in lipid peroxidation (32.8%), and decreased disease severity (80%).

**Conclusion:**

These findings suggest that *Aspergillus fumigatus* and *Curvularia lunata* induce the anti-pathogenic metabolites, defense-related protein, antioxidant enzymes, resistance genes, salicylic acid (SA), and jasmonic acid (JA) biosynthesis. Together, these responses enhance the overall defensive capacity of wheat against stripe rust, providing a sustainable and ecologically friendly alternative to synthetic fungicides for controlling wheat stripe rust.

## 1 Introduction

*Puccinia striiformis* Westend. f. sp. *tritici* (Pst) producing stripe rust, often identified as yellow rust, is a very severe and harmful infection that affects the wheat plant worldwide, particularly in colder and more humid climates (Khanfri et al., 2018; Ashraf, 2024). The Pst is a severe threat because its urediniospores can spread by wind over long distances, allowing the pathogen to cause extensive yield damage and widespread epidemics in favorable environmental conditions (Brown and Hovmøller, 2002; Hovmøller et al., 2002). Kernel shriveling can result in substantial quality and yield injuries of over 75% during severe outbreaks (Conner and Kuzyk, 1988).

*Triticum aestivum* (bread wheat) is one of the important crops grown on 216.7 million hectares worldwide. Bread wheat is a cereal grain containing high-protein content that delivers 20% of total calories, enough for feeding 30% of the world’s population, and is a rich source of vitamins (E, B1, B2, and B3) and minerals (Mn, P, Cu, and Se) (George et al., 2024). It accounts for 21% of the world’s food demand and is cultivated on approximately 200 Million Hectares of farmland worldwide (Jasrotia et al., 2018). Wheat sets up nearly 1/5th of the overall calorie consumption of the world’s inhabitants. It is estimated that 82–85% of the world’s population relies on wheat for essential calories and protein, respectively (Caverzan et al., 2016). In Pakistan, wheat is one of the basic diets and is grown nationwide during the winter season (Sajid Ali et al., 2009). Pests and pathogens, the natural factors that cause major damage to wheat crops, result in less production of yield depending on the severity of the disease and the environmental conditions (Wang and Chen, 2013). Instead, the increasing growth in population is creating grave warnings about food safety. Moreover, the other issues include outdated agricultural approaches and a lack of scientific results that can challenge agricultural procedures. As per the previous financial year, the COVID-19 epidemic has destructively affected crop development. Hence, growing the production of wheat is expected to promise food protection (Shafi et al., 2022).

The Pst is a vital wheat infection in many countries, including Pakistan (Ali et al., 2014a). The wheat rusts are grouped into three distinct sets: stripe rust produced by *P. striiformis* f. sp. *tritici* (Pst), stem rust initiated by *P. graminis* f. sp. *tritici* (Pgt), and leaf rust caused by *Puccinia triticina* (Pt). These rusts together cause a substantial reduction in wheat production. Similarly, in Pakistan, like other countries, the wheat stripe rust causes a huge reduction in the wheat total yield (Ali et al., 2014b). Pst is a biotroph that belongs to the phylum Basidiomycota; only the green and fleshy tissue of grasses and cereals is infested by the pathogen (Dean et al., 2012). Once the signs appear, the spore formation occurs after 2–3 weeks of leaf contamination under optimal temperature. Stripe rust is largely initiated in 70% of the wheat-growing region in Pakistan (Singh et al., 2004).

Plants respond to biotic stresses in the natural environment by developing defense mechanisms triggered by biocontrol agents. Therefore, to exploit their secondary metabolites, the use of biocontrol agents may be a good approach. The systemic acquired resistance (SAR) and the induced systemic resistance (ISR) are the two central autoimmune systems involved in these reactions. In contrast to the well-established mechanism of SAR triggered by a pathogen dose, certain biocontrol agents regulate phytopathogens through indirect interactions by activating the ISR (Conrath, 2011).

*Trichoderma* (*T. harzianum* and *T. asperellum*) are recognized biocontrol agents, utilized worldwide since they generate various antimicrobial secondary metabolites. *Trichoderma* sp. is said to (a) be an efficient biocontrol agent against fungal phytopathogens, (b) be an excellent growth stimulator and biofertilizer, (c) cause biotic resistance and abiotic stress, and (d) release chemical elicitors to promote a plant response to stress (Khan et al., 2020). Metabolites secreted by *Trichoderma* contribute to the biological regulation of infections. *Trichoderma* sp. has proven anti-phytopathogenic efficacy by generating a variety of antifungal, proteinaceous elicitors and secondary metabolites. *Trichoderma* has moreover been shown to stimulate the ISR by modulating defense-associated gene expression, for instance, Sm1 (*T. virens*) triggered the ISR in maize (Abd El-Rahman et al., 2014). Through the induction of defense-related genes, the *T. harzianum* (Epl-1) protein has also been identified to regulate *Botrytis cinerea* contamination in tomatoes (Gomes et al., 2017).

The universal protection system in plants is incompetent to alleviate stress and to handle the complexities of different combined stresses that occur in plants. Thus far, genetic approaches and additional chemical and physical practices have been utilized to grow stress-tolerant cultivars. Subsequently, they cannot afford to overcome stress for a lengthy time and are not ecological. Thus, connecting the potential of valuable endophytes existing in the environment for infection control could be a substitute tactic for successful plant struggle and flexibility in crop selections (Pan et al., 2022).

Plant-pathogen interactions are categorized by the presence of pathogen-related (*PR*) proteins, which are represented by a broad collection of 17 protein families. There are many types of *PR* proteins, having different functions, including *PR1* (which has an unknown function). *PR2* function is to break down 1,3-glucans, *PR3*, 4, 8, and 11 break down chitin wall in fungus, thaumatin-like proteins (*PR5*) that disrupt fungal cells, proteinase inhibitors (*PR6*) to block pest and pathogens, proteinase (*PR7*) break down harmful proteins, peroxidase (*PR9*) produce defensive compounds, ribonuclease like proteins (*PR10*) that degrade pathogen RNA, defensins (PR12) disrupts microbial membrane, thionins (*PR13*) and *PR17* with unknown function (Wu et al., 2025). Many of these *PR* proteins showed function in plant defense against pathogens, but their involvement against wheat-Pst still needs to be further investigated. Several resistance genes have been identified that are effective in controlling yellow rust, but many of these genes have been overcome by pathogen populations. Only *Yr5, Yr15*, some temporarily designated genes, and several adult-plant resistance genes are still effective against stripe rust in China. The *Yr5* and *Yr15* have been reported to have complete resistance to all races in California (Chugunkova et al., 2025). Many yellow rust resistance gene like *Yr5* and *Yr10* are absent in Morocco wheat variety. Due to the absence of these marker genes, they are highly susceptible to yellow rust (Din et al., 2023).

Microorganisms known as endophytes are readily isolated from the inside of plants or from surfaces that have been cleaned. Furthermore, they are the best candidates for biocontrol agents due to their ecological niche, similar to phytopathogens. Rust infections have been controlled with the application of microorganisms (El-Sharkawy et al., 2018). While endophytic bacteria have historically been employed to manage rust infections, the biological control of Pst was first reported in 2010 (Kiani et al., 2021). Recent studies indicate that *Arbuscular mycorrhizal* (AM) fungi and *Trichoderma* sp. diminish the prevalence of wheat stem rust (El-Sharkawy et al., 2018). Two native endophytic fungi isolated from the roots of the *Phoenix dactylifera* plant were investigated in the current study for their potential use as a biocontrol agent against stripe rust in wheat plants.

## 2 Materials and methods

### 2.1 Isolation of endophytic fungi from *Phoenix dactylifera* root

*Phoenix dactylifera* L. root samples were obtained from Takht Bhai, a small tehsil of District Mardan in Khyber Pakhtunkhwa province of Pakistan, having 34° 05’ to 34° 32’ North latitudes and 71° 48’ to 72° 25’ East longitudes. The samples were treated with 70% ethanol for 1 min and 2.0% NaCl for 2 min, followed by thorough washing with autoclaved distilled water. The roots were cut into small sections by a sterile razor blade, spread on Hagem minimal medium, and incubated at 25 °C for 7 days. Emerging colonies from the root sections were re-cultivated on potato dextrose agar (PDA) medium at 28°C for 1 week. The isolated colonies were cultured in Czapek culture broth liquid medium (30°C, 120 rpm, 7 days). After the culturing period, the fungal filtrate and biomass were separated using filter paper and stored for further biochemical and metabolic analyses, as described previously (Rahman et al., 2023). The fungal biomass was washed twice with distilled water and dried at 70°C till it reached a constant weight (Saad et al., 2019).

### 2.2 Fungal isolates characterization for plant growth-stimulating traits

The gibberellic acid (GA) and indole-3-acetic acid (IAA) quantities were observed by the method described by Fernando et al. (2021). The supernatant (1 mL) was mixed with 5 mL combined solution (chloroform, methanol, and ammonium hydroxide) (i.e., 12:5:3 v/v/v) and 25 mL dH_2_O. The chloroform level was discarded, while the H_2_O-CH_3_OH level was evaporated. The ethyl acetate (15–20 mL) was then added and kept warm in incubation for 1 h at 70°C. The ethyl acetate part was evaporated at 45°C, and ODs were recorded for GA (254 nm) and IAA (280 nm). For the quantification of proline, supernatant (2 mL) was mixed with acid-ninhydrin reagent (2 mL). The ninhydrin (1.2 g) was added to sulphuric acid (20 mL) and glacial acetic acid (30 mL) to make an acid-ninhydrin reagent. The mixture was heated for an hour in a water bath, and toluene (4 mL) was added at the end. With the help of a dropper, toluene layers were separated. The optical density was measured at 520 nm, as previously reported by Khalafallah and Abo-Ghalia (2008). The flavonoids were quantified by following the protocol reported previously by Upadhyaya et al. (2010), where the supernatant (0.5 mL) was added to ethanol (5 mL of 80%), and shaken vigorously for one day constantly (120 rpm). The potassium acetate (0.1 mL), 80% methanol (4.3 mL), and 10% aluminum chloride (0.1 mL) were combined. After 30 min of incubation in the dark, the OD was recorded at 415 nm. The phenolics were identified using the protocol of Ribeiro et al. (2007), with slight modifications. The distilled water (3 mL) and supernatant (2 mL), collected after centrifugation of the plant sample, were mixed. Then, the Folin-Ciocalteu reagent (0.5 mL) and 20% sodium carbonate (3 mL) were added. The multiple redox responses between phosphor-molybdic acid and phenols produced the molybdenum blue complex. After warming for 30 s, the complex was cooled down, and the OD was recorded (650 nm). The DPPH scavenging activity was done by following the protocol reported previously by Abbasi et al. (2016). Fungal culture filtrate (FCF) (0.1 mL) was added to methanol (1 mL). For making a stock solution, 0.004% DPPH was added to methanol (100 mL). The diluted sample (0.5 mL) combined with DPPH (1 mL) was incubated in full darkness for 60 s, and the OD was recorded (517 nm) through a color change.

### 2.3 Compatibility bioassay of isolates in the dual-culture approach

The isolates were unable to inhibit the growth of mycelium and spore formation, suggesting their compatibility in a dual-culture experiment as described earlier by Rahman et al. (2023). The compatibility and antagonistic interactions of DT-4 and DT-8 isolates were evaluated on PDA nutritional medium plates in relation to one another. The DT-4 and DT-8 samples, each containing 1 × 10^∧^7 spores/mL, were injected with a total volume of 20 μL at the middle of the PDA plates. The experiment was conducted in triplicate, and the incubation was maintained at 26 ± 2°C.

### 2.4 Molecular identification of fungal isolates

Total DNA was extracted with the help of a DNAeasy plant micro kit (QIAGEN et al., United States) following manufacturer instructions with slight modification (Zhang et al., 2010). The ITS region of 18S rRNA was amplified by PCR with the help of unique primers ITS1 (5’-TCCGTAGGTGAACCTGCGG-3’) and ITS4 (5’-GCTGCGTTCTTCATCGATGC-3’) and sent for custom sequencing (BGI, Shanghai, China) to check the identity of endophytic fungi (Ali et al., 2022). The sequences were subjected to NCBI for homology search, and trees of the neighbor-joining (NJ) were constructed by using MEGA 11.

### 2.5 Effect of DT-4 and DT-8 isolates on vegetative growth of wheat

A total of 10 distinctly purified strains were selected for preliminary screening to assess the growth-promoting potential on wheat seedlings. The biomass was prepared from the purified fungal isolates, which were upscaled using Czapek culture broth liquid media (30°C, 120 rpm, 7 days). A pellet of fungal biomass and supernatant were separated and stored at -70°C to conduct biochemical and metabolic analysis. The fresh biomass from the growing media was used to soak surface-sterilized wheat seeds. The inoculated seeds were allowed to air dry under the laminar flow hood cabinet. The same conditions were used to soak control seeds in 1 mL of sterile water. The inoculated and control seeds were then put on wet filter paper with a 75% humidity level and a temperature range of 18–28°C for germination.

### 2.6 Morphological and molecular identification of Pst strain

The Pst fungal strains isolated from infested wheat leaves displayed distinctive signs of yellow rust infection. The spores were maintained by spreading them on the leaves of vigorous wheat seedlings of the Morocco variety that is highly susceptible to yellow rust in the laboratory. The leaves of 10-weeks old seedlings were retained on damp sheet towels in acrylic boxes. The abaxial side of the leaves was inoculated with conidial suspension (Zhang et al., 2024). A light microscope was used to see mycelium at magnifications of 40× and 100× to examine the morphological features of hyphae, conidiophores, and conidia. Molecular identification of the pathogenic fungus was done as mentioned in section 2.3.

### 2.7 Preparation of strip rust spore suspension

As an unculturable fungus, Pst could not be developed on non-natural nutrient media. To collect sporangia, the confirmed diseased leaves were obtained from wheat plants infected by the yellow rust infection under greenhouse conditions. The spores were obtained from the lesion regions of infested leaves by spraying the sterilized dH_2_O mixed with Triton X-100 (0.01 %). The spore suspension was centrifuged for 15 min at 10,000 rpm, and the pellet was cleaned 3 times with sterilized dH_2_O. After 30 s of vortexing, the pellet was re-suspended in sterilized dH2O at a final concentration of 1 × 10^8 spores/mL (Sørensen et al., 2016). A hemocytometer was used to observe the spore suspension.

### 2.8 Anti-pathogenic bioassay for whole detached leaf

Healthy and vigorous leaves were removed at the 3rd–6th node of the wheat shoot and cleaned. The leaves were soaked in the disinfected petri plates and spread on a filter paper (Whatman No. 1/5.5 cm) already soaked in a growth medium (2 mL) (Rauf et al., 2022). The leaf was incubated (30 °C for 24 h dark) followed by infection with the freshly set SR-S (1 × 10^8 spores/mL). After 36 h, when stripe rust symptoms started to show, the leaves were inoculated with DT-4, DT-8, and DT-4+DT-8 (1 × 10^8 spores/mL) using the same concentration of inoculum. Petri plates were sealed and again incubated at 21/18 °C under a 12/12 h light/dark cycle. The whole detached leaf bioassay was performed thrice, independently as individual biological replicates, with six technical replicates of each biological replicate, for each treatment.

### 2.9 Application of DT-4 and DT-8 isolates for plant-germination and growth bioassay

The TD-1 and Morocco wheat varieties, which are highly susceptible to stripe rust (Khalifa et al., 2024), were provided by the Cereal Crops Research Institute (CCRI), Pirsabak, Nowshera, KP, Pakistan. Physical appearance was used to choose mature, healthy, and uniformly proportioned seeds. Autoclaved distilled water was used to wash the seeds three times. The wheat seeds were sterilized using 70% ethanol followed by three rounds of washing with distilled water. The seeds were planted in DT-4 and DT-8 biomass mixed soil (2 g/100 g of soil) in the pots and shifted to a growth chamber (day/night cycle: 14 h at 28°C ± 0.3°C, 10 h at 25°C ± 0.3°C; relative humidity 70%; six plants per treatment) in the laboratory at Abdul Wali Khan University, Mardan. A total of eight treatments with six repetitions for each were used in a fully randomized manner.

*Experimental design:*

Treatment No 1. Control
Treatment No 2. DT-4
Treatment No 3. DT-8
Treatment No 4. DT-4+DT8
Treatment No 5. SR-S
Treatment No 6. SR-S+DT-4
Treatment No 7. SR-S+DT-8
Treatment No 8. SR-S+DT-4+DT8

### 2.10 Plant materials and condition for anti-pathogen plant bioassay

Cultures of DT-4 and DT-8 fungal endophytes having anti-pathogenic activity against Pst were inoculated into plastic pots containing autoclaved soil. The seeds of both varieties, TD-1 and Morocco, were treated with FCF of both strains individually and combined. The cold SR-S was applied to 90-days old plants as a spray. After 36 h, the appearance of the disease, the DT-4 and DT-8 endophyte filtrates were inoculated onto the plants, which were then shifted to a growth compartment (12-h day length at 23°C and a 12-h night with 60–70% relative humidity).

### 2.11 Agronomic data

The data were verified for several agronomic/yield-associated parameters, along with the data for yellow rust. These traits involved days to maturity, days to heading, tillers m^–2^, spike weight (g), grain weight spike^–1^, plant height, and grains spike^–1^.

### 2.12 Yellow rust scoring

As shown in Table 1, the disease severity level was determined by following the protocol of Hayıt et al. (2021).

**TABLE 1:** Disease scoring of both varieties.

Reaction	Observation	Response value
No infection/disease	O	0.0
Unaffected/resistant	U/R	0.2
Mildly resilient/ resistant	MR	0.4
Mildly resistant- mildly susceptible	MR-MS/M	0.6
Mildly susceptible	MS	0.8
Susceptible	S	1.0

### 2.13 Histochemical recognition of H_2_O_2_

For detection of the H_2_O_2_ production and accumulation with DAB (3,30-diaminobenzidine; Sigma, St. Louis, MO, United States), wheat leaf tissues were treated with DAB staining solution (1 mg_mL^1^) and were kept for 8 h. Tissues then were boiled for 20 min in ethanol/lactic acid/glycerol (3:1:1 ratio) and transferred to ethanol (95%) at 4**°**C for the store. The accumulation of H_2_O_2_ was assessed by the appearance of a brown color due to the polymerization of DAB. A digital camera was used to take images of the leaf segments (Kumar et al., 2014).

### 2.14 Quantification of antioxidant enzymes

The enzymatic activity in plant tissue was measured by a spectrophotometer 10 days after the treatment with endophytes. The oxidation of NADPH was used to measure reduced glutathione (GSH) activity as described previously (Keutgen and Pawelzik, 2007). The activity of peroxidase (POD) was determined by following the previously reported method (Gorin and Heidema, 1976). Following the reported method of Amako et al. (1994), the catalase (CAT) activity and ascorbate peroxidase (APX) activity were determined by oxidizing ascorbic acid.

To determine the MDA concentration, the well-crushed leaf tissue section (0.5 g) was made uniform with thiobarbituric acid or TBA (3 mL of 0.6%), centrifuged (12,000 rpm for 10 min), heated in a water bath (10 min), chilled, and centrifuged again (10 min at 12,000 rpm). The absorbance measurements were made at 450, 600, and 532 nm (Isgandarova et al., 2024). Catalase activity was evaluated by H_2_O_2_ cleavage, which involved adding 400 μL of 3% H_2_O_2_, supernatant (0.1 mL), pH seven phosphate buffers (2.6 mL), and EDTA (0.1 mM) (Hussain et al., 2025). The OD was measured by 240 nm. Furthermore, the content of superoxide dismutase (SOD) was measured at 580 nm by following the method described previously (Khan et al., 2021).

### 2.15 Chlorophyll pigments

The fresh leaves (0.5 g) were taken 10 days post inoculation with endophytes and ground in acetone (2 mL of 80%). After extraction, the samples were centrifuged at 1,500 rpm for 10–20 min. The supernatants were analyzed for chlorophyll pigments. For chlorophyll a, b, and total carotenoids, the ODs were recorded at 645, 663, and 480 nm, respectively (Arnon and Whatley, 1949). Evaluation of chlorophyll contents was done by following equations:

Chlorophyll “a” content (mg/g F W) = (12.8 × A663) – (2.8 × A645)
Chlorophyll “b” content (mg/g F W) = (22.9 × A645) – (4.67 × A663)
Carotenoid’s content = A 480 + (0.114 × A 663 – 0.637 × A 645)
Total chlorophyll content (mg/g F W) = (0.0202 × A645) + (0.00802 × A663)

### 2.16 Evaluation of secondary metabolites

As mentioned, the total flavonoid content was determined by the AlCl_3_ method (Maclachlan and Zalik, 1963) 10 days after the endophytes treatment. The phenolic content was assessed using the method discussed. Prolines were assessed by the protocol of Bates et al. (1973). The total soluble sugar assessment was done by the protocol of Mohammadkhani and Heidari (2008). The method described by Song et al. (2010) was followed to measure the total phenolic contents.

### 2.17 Phytohormonal analysis

In order to measure the endogenous levels of salicylic acid (SA) and jasmonic acid (JA) from each condition, leaves from the fourth and sixth nodes of the wheat plant were removed after 10 days post inoculation with endophytes DT-4 and DT-8, and stored at –80.

Total SA content was assayed as reported previously by Mehdi et al. (2025) and Horchani et al. (2025). With the help of methanol (1 mL; 90% v/v), 150 mg of frozen leaf tissue was powdered, followed by vortexing, sonication (3 min), and centrifugation (10,000 × g/10 min at room temperature) to obtain supernatant. The pellet was resuspended in 100% Methanol (100% v/v; 0.5 mL) for re-extraction as ‘mentioned above. The pellets were resuspended in TCA (0.25 mL; 5% w/v), after the evaporation of the supernatant (40 °C/vacuum), and partitioning was done by adding ethyl acetate/cyclohexane (1:1 v/v; 0.8 mL). Vacuum concentration of free-SA content from the upper phase was done at 40°C. HCl (8 M; 0.3 mL) was added to the conjugated SA in a lower phase for hydrolysis, and incubated at 80°C for 60 min. Total combined SA was dissolved in methanol and quantification of SA was done in a mobile phase by taking absorbances at 305 and 407 nm with the flow rate (0.8 mL/min).

Similarly, JA content was quantified by homogenizing the frozen leaf tissue with ethyl acetate (1 mL) and incubating overnight (4°C). The supernatant was harvested by centrifugation (10,000 × g/10 min/4°C) and mixed with acidified water (0.2% v/v), and the separated aqueous phase was analyzed with HPLC. JA was quantified by taking absorbance at 210 nm with the flow rate of 1 mL/min (Mehdi et al., 2025).

### 2.18 Gene expression quantification by RT-qPCR in wheat

Total RNA was extracted from 150 mg of frozen leaf tissue using the MiniBEST Plant RNA Extraction Kit (TaKaRa, Dalian, China). The integrity of RNA samples (260/280 nm absorbance ratio of 1.8–2.0) was assessed by agarose gel (1%) electrophoresis. Revert Aid First Strand cDNA Synthesis Kit by Invitrogen (Karlsruhe, Germany) was used to prepare cDNA from DNase-treated RNA (2 μg). Gene expression analysis of pathogenesis-related protein genes *PR1* and *PR5* was conducted using primers listed in Table 2.

**TABLE 2:** qRT-PCR primers for gene expression of *PR1* and *PR5* genes.

Primer name	Forward primer	Reverse primer	References
*PR1*	CTGGAGCACGAAGCTGCAG	CGAGTGCTGGAGCTTGCA GT	(Esmail et al., 2022)
*PR5*	ACAGCTACGCCAAGGACGAC	CGCGTCCTAATCTAAGGGCAG	(Duan et al., 2014)

### 2.19 Statistical analysis

The experiments were repeated a minimum of three times. The data were analyzed using SPSS software. Means were matched by ANOVA and Duncan’s multiple range tests (*P* = 0.05).

## 3 Results

### 3.1 Morphological and phylogenetic characterization of fungal isolates

**FIGURE 1:**
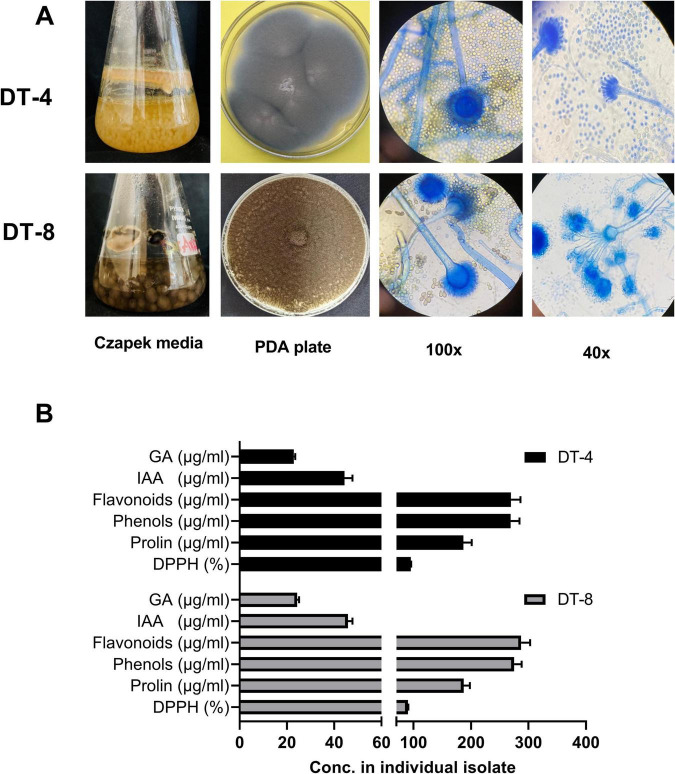
Characterization of fungal isolates. **(A)** Morphological characterization of DT-4 and DT-8 isolates. Images are taken at 100× and 40× magnification. **(B)** Quantification of metabolites in fungal culture filtrates (FCF).

The physiological characteristics of DT-4 and DT-8 fungal isolates were assessed using liquid and solid media (Figure 1A). DT-4 exhibited healthy growth in the Czapek medium, with a dense yellowish appearance, and on PDA plates, it formed compact colonies with pale pigmentation. Microscopic examination under 40x and 100x magnifications revealed well-defined hyphal structures with characteristic spore formations. Similarly, DT-8 displayed vigorous growth in the Czapek medium, with dark pigmentation, and on PDA plates, it produced dense, brown-colored colonies. Microscopic analysis showed distinct hyphal branching and spore morphology, indicative of its unique structural features. The quantitative analysis of phytohormones and antioxidant activity of DT-4 and DT-8 is presented in Figure 1B and Supplementary Table 1. Both fungal isolates produced similar levels of gibberellic acid, indole-3-acetic acid, flavonoids, phenols, and proline. The molecular characterization of two fungal endophytes was performed through phylogenetic analysis, as shown in Supplementary Figure 1. The sequence homology of DT-4 appeared with *Curvularia lunata* (95 %) and DT-8 with *Aspergillus fumigatus* (100 %). The sequences were deposited to NCBI Gene Bank under accession numbers PX323530 and PX316415.

### 3.2 Effect of endophytic fungi on wheat seed germination

**FIGURE 2:**
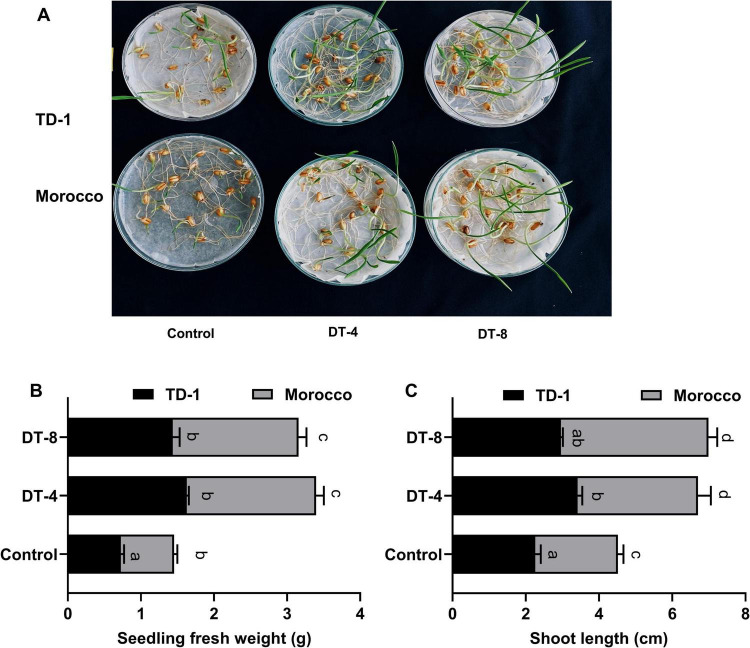
Effect of fungal culture filtrates (FCF) on wheat germination: **(A)** effect of FCF on seed germination, shown 7 days post germination, **(B)** seedling fresh weight, **(C)** shoot length 14 days post germination.

The germination assay demonstrated a significantly favorable influence of fungal isolates DT-4 and DT-8 on wheat seeds compared to the control group (Figure 2A). Seeds treated with DT-4 and DT-8 exhibited enhanced germination rates, with visible seedling vigor and increased shoot development. The fresh weight of the seedlings was considerably higher in the fungal-treated groups compared to the control (Figure 2B). Seedlings treated with DT-4 exhibited the most significant increase in fresh weight, 3.39 g for Morocco and 1.62 g for TD-1, followed closely by DT-8, 3.15 g for Morocco and 1.62 g for TD-1. In contrast, control seedlings showed the lowest weight, 1.5 g for Morocco and 0.72 g for TD-1. This indicates the potential of these fungal isolates to improve biomass accumulation. Shoot length was markedly enhanced in fungal-treated groups, as shown in Figure 2C. In the TD-1 wheat variety treatment, DT-4 increased shoot length to 3.41 cm, representing a 51.6% increase, while treatment DT-8 increased to 2.96 cm, corresponding to a 31.6% increase as compared to the control (2.25 cm), shown in Supplementary Figure 2. Similarly, for the Morocco variety, DT-4 treatment increased to 6.70 cm, corresponding to a 48.6% increase, whereas treatment DT-8 increased to 6.90 cm, representing a 53.0% increase as compared to the control of 4.51 cm. These results highlight the role of DT-4 and DT-8 in promoting wheat seedling growth through elongation of the shoot system.

### 3.3 Screening for the antipathogenic fungal endophytes using a whole detached wheat leaf bioassay

**FIGURE 3:**
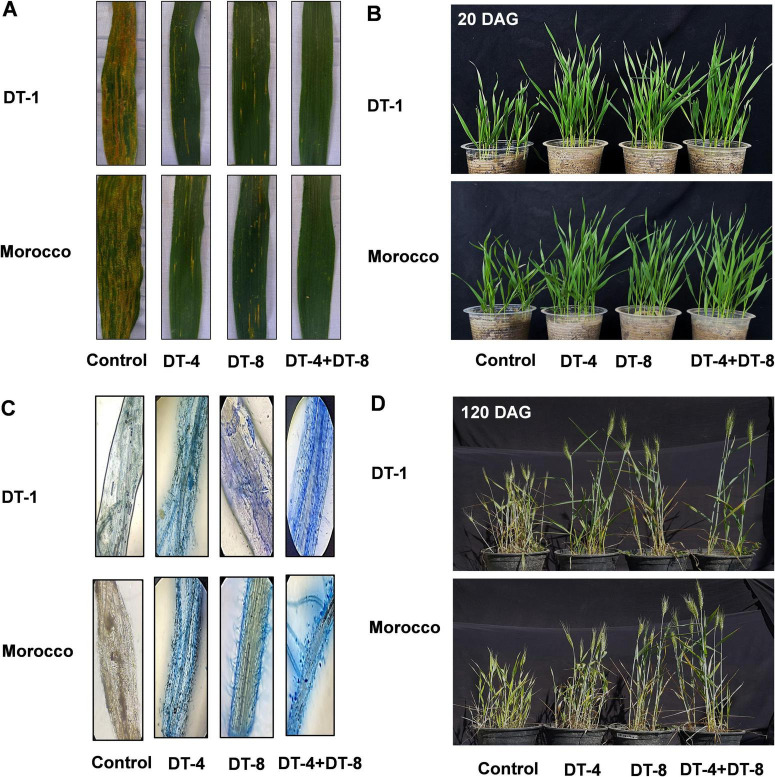
Phenotypic examination of wheat plants related to endophytic fungi (DT-4 and DT-8). **(A)** Whole detached leaf bioassay for anti-pathogenic evaluation of endophytes (leaves infected with SR-S and inoculated with DT-4 and DT-8 isolates for TD-1 and Morocco variety). **(B)** 21 days plants inoculated with DT-4 and DT-8 isolates. **(C)** Root colonization visuals in 20 days old plants, and **(D)** 120 days old plants (30 dpi with stripe rust) pre-inoculated with DT-4 and DT-8 isolates for TD-1 and Morocco variety.

Using a detached whole-leaf bioassay, the initial impact of the fungal isolates (DT-4 and DT-8) and pathogen SR-S co-inoculation in wheat was examined, allowing for the evaluation of disease severity on wheat leaves. The visual observations of disease symptoms on leaves under different treatments are presented in Figure 3A for TD-1 and Morocco. The untreated wheat leaves exhibited extensive yellow rust infection with prominent chlorosis and necrosis, indicating severe disease progression. DT-4 and DT-8, individually, displayed a moderate reduction in disease symptoms compared to the control. The leaves showed less chlorosis and necrosis, suggesting partial inhibition of Pst. These results highlight the potential of DT-4 and DT-8 as biocontrol agents against. Furthermore, the combined application of DT-4 and DT-8 exhibited the most potent suppression of disease symptoms (Figure 3A).

### 3.4 Effect of fungal endophytes on wheat plant growth

Wheat plants treated with fungal endophytes (DT-4, DT-8, and DT-4+DT-8) showed significantly enhanced growth compared to controls, both at the early (21 days) and later stage (120 days) under SR-S infection, as shown in Figures 3B,D. Microscopic analysis confirmed the presence of stripe rust spores and infected wheat leaves displaying characteristic yellow streaks. Plants treated with combined biocontrol agents (DT-4+DT-8) or their integration with stripe rust (DT-4+DT-8+SR-S) exhibited improved health and reduced pathogen impact compared to the control. Root colonization analysis revealed substantial fungal colonization in DT-4 and DT-8 treatments, indicating their role in promoting plant growth and mitigating pathogen-induced stress (Figure 3C).

### 3.5 Determination of chlorophyll contents in wheat inoculated with DT-4 and DT-8

**FIGURE 4:**
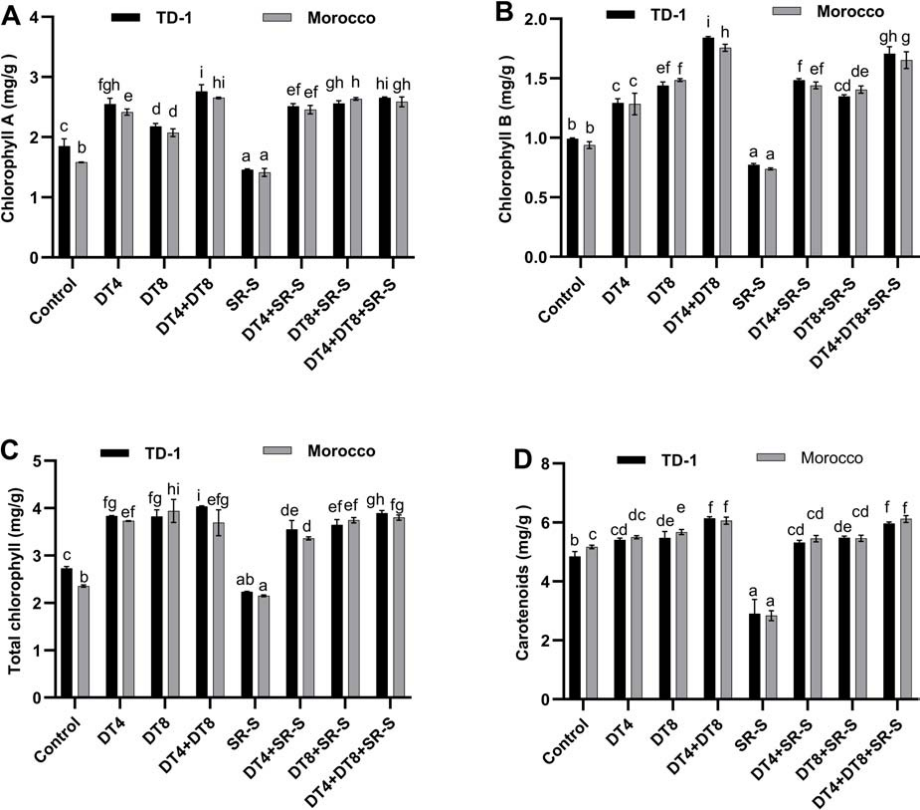
Photosynthetic pigments in wheat: photosynthetic pigments in 120 days old plants (30 dpi with stripe rust) pre-inoculated with DT-4 and DT-8 isolates for TD-1 and Morocco variety, **(A)** chlorophyll A, **(B)** chlorophyll B, **(C)** total chlorophyll, **(D)** carotenoids. The quantitative data are presented as means ± SE, with various letters indicating significant differences (*p* ≤ 0.05).

Plants treated with fungal isolates under both normal and pathogen-treated conditions demonstrated a significant increase in chlorophyll, as indicated by quantitative data. Figures 4A,B illustrates how the isolates (DT-4 and DT-8) treatment restored the chlorophyll content in comparison to untreated plants, whereas the pathogen decreased it. Under control conditions, the total chlorophyll content was 2.7 mg/g in TD-1 and 2.3 mg/g in Morocco. However, treatment with DT-4 and DT-8 individually increased total chlorophyll to 3.8 mg/g (TD-1), 3.7 mg/g in (Morocco), and 3.8 mg/g (TD-1), 3.9 mg/g (Morocco), respectively. Moreover, combined inoculation of DT-4+DT-8 significantly (*p* ≤ 0.05) elevated chlorophyll content in both TD-1 (4.0 mg/g) and Morocco (3.6 mg/g) varieties in comparison to the control treatment. Chlorophyll content decreased after SR-S infection to 2.2 mg/g in TD-1 (–20%) and 2.1 mg/g (–7%) in Morocco. However, with the addition of DT-4, it is significantly restored to 3.5 mg/g (+29.63%) in TD-1 and 3.3 mg/g (+43.48%) in Morocco. Moreover, DT-4+DT-8+SR-S further enhanced it to 3.8 mg/g (+40.74%) in TD-1 and 3.82 mg/g (+65.22%) in Morocco, as illustrated in Figure 4C. Similar trends were seen in carotenoids, where SR-S infection reduced chlorophyll levels in wheat leaves, while the combined treatment of DT-4+DT-8 with and without SR-S recovered carotenoids significantly, as shown in Figure 4D.

### 3.6 Growth-related metabolites in wheat inoculated with DT-4 and DT-8 and stripe rust

**FIGURE 5:**
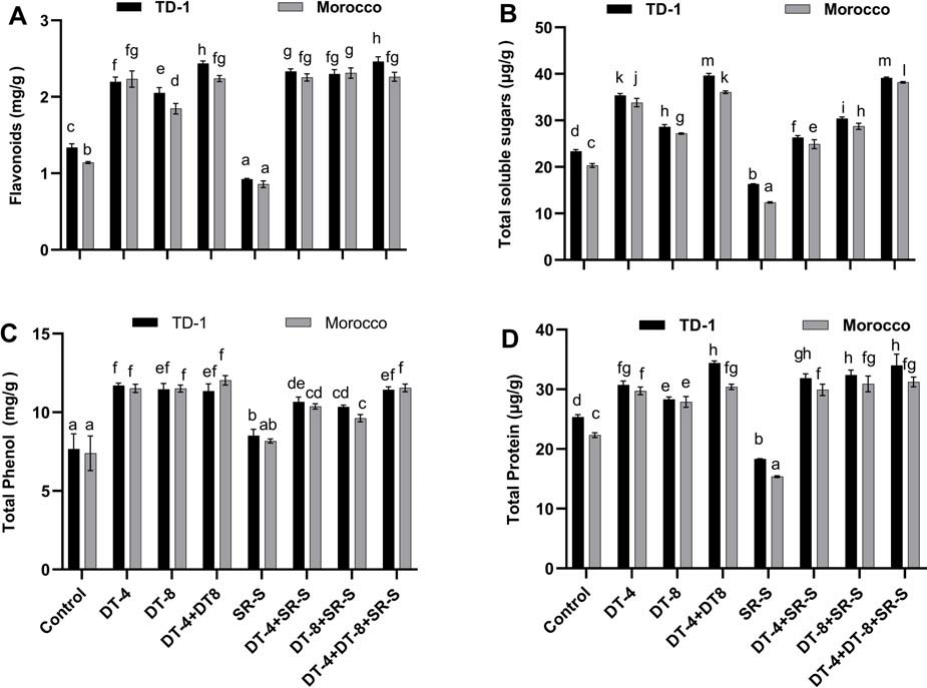
Growth-promoting metabolites in wheat: growth-promoting metabolites in 120-day-old plants (30 dpi with stripe rust) pre-inoculated with DT-4 and DT-8 isolates for TD-1 and Morocco variety, **(A)** flavonoids, **(B)** total soluble sugars, **(C)** total phenolics, **(D)** total protein content. The quantitative data display means ± SE, with different letters representing significant differences (*p* ≤ 0.05).

The effect of DT-4 and DT-8 endophytes on growth-related metabolites of wheat with and without SR-S infection was also assessed. Both DT-4 and DT-8 individual inoculation elevated the levels of flavonoids, total soluble sugars, total phenols, and total proteins in wheat, with the changes being more significant when treated with both strains, DT-4 and DT-8 (Figure 5). After infection with the SR-S, the flavonoids dropped to 0.92 mg/g (-30.83%) in TD-1 and 0.85 mg/g (-25.44%) in Morocco compared to the untreated control, and were restored significantly by the addition of DT-4 and DT-8 individually and in combination (+85.0% TD-1, +98.25% Morocco) as shown in Figure 5A. A similar trend was noted while measuring soluble sugar content. Under the control condition, the soluble sugar level increased significantly with the treatment of DT-4 (+52.17% TD-1, +65.00% Morocco) and DT-8 (+21.74% TD-1, +35.00% Morocco) individually, and further enhanced by the combined treatment of DT-4+DT-8 (+69.57% TD-1, +80.00% Morocco) compared to control. The SR-S infection reduced the sugar level in both TD-1 (–30.43%) and Morocco (–40.00%) varieties compared to the untreated control, while restoring it by the treatment of DT-4, DT-8, and DT-4+DT-8 (+69.57% TD-1, +90% Morocco) as illustrated in Figure 5B. Total Phenol content was reduced with SR-S infection (+11.84% TD-1, +10.96% Morocco) as compared to the control, and recovered when the plants were treated individually with DT-4, DT-8, while a further significant increase was noted with the combined treatment of DT-4+DT-8 (+50.00% TD-1,+57.53% Morocco) was observed (Figure 5C). Similarly, total protein was reduced when plants were infected with SR-S (-28.00% TD-1, -31.82% Morocco). The reduction was recovered significantly (*p* ≤ 0.05) by the individual and combined treatment of DT-4 and DT-8 strain (+32.00% TD-1, +40.91% Morocco) as shown in Figure 5D. Quantitative data are represented as mean ± SE, with various letters indicating significant differences (*p* ≤ 0.05) according to the Duncan test, as shown in Supplementary Table 2.

### 3.7 Antioxidant potential in wheat inoculated with DT-4 and DT-8 under SR-S infection

**FIGURE 6:**
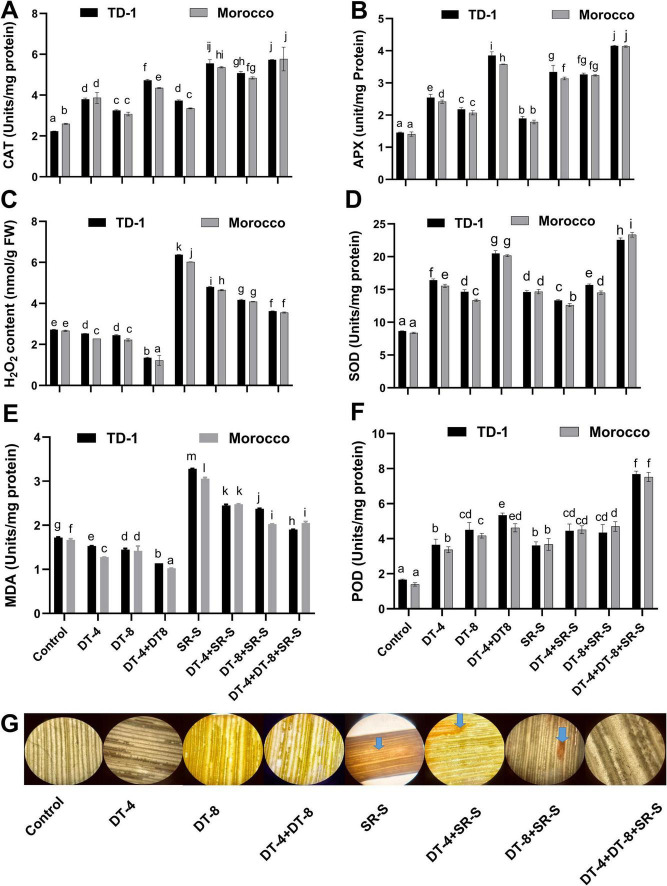
Antioxidant potential in wheats: antioxidant potential in 120 days old plants (30 dpi with stripe rust) pre-inoculated with DT-4 and DT-8 isolates for TD-1 and Morocco variety, **(A)** catalase activity, **(B)** ascorbate peroxidase, **(C)** H_2_O_2_ content, **(D)** superoxide dismutase, **(E)** malondialdehyde content, **(F)** peroxidase, **(G)** DAB staining images were taken at 40x magnification. The quantitative data show means ± SE, with different letters representing significant differences *(p* ≤ 0.05).

The antioxidant enzyme activities (CAT, APX, SOD, POD) were also affected upon DT-4 and DT-8 inoculation in wheat infected with and without SR-S. Under the SR-S infection, individual inoculation of DT-4 and DT-8 significantly (*p* ≤ 0.05) induced the enzyme activities compared to the control. However, DT-4 and DT-8 strain co-inoculation resulted in a further increase in the antioxidant enzyme activities of CAT (+156.50% DT-1, +122.3% Morocco) (Figure 6A), APX (+186.21% DT-1, +192.91% Morocco) (Figure 6B), SOD (22.56 Units/mg proteins in TD-1 and 23.35 Units/mg proteins in Morocco) (Figure 6D) and POD (+362.65% TD-1, +440.29% Morocco (Figure 6F).

Furthermore, the level of H_2_O_2_ was enhanced in the plant infected with SR-S and was significantly reduced after the co-inoculation of DT-4 and DT-8 strain (–45.46% TD-1, –41.03% Morocco), as compared to the SR-S treated (Figure 6C). A similar increasing trend was observed in the MDA level when infected with SR-S, and was significantly reduced with the co-inoculation of DT-4 and DT-8 strain (–42.07% TD-1, –32.79% Morocco) as compared to SR-S (Figure 6E). Moreover, DAB staining revealed increased H_2_O_2_ accumulation under SR-S infected, evidenced by dark staining in leaves compared to the minimal staining showed in the control. DT-4 and DT-8 reduced staining intensity compared to SR-S, which is further reduced with DT-4+DT-8+SR-S treatment, indicating reduced oxidative stress (Figure 6G). Quantitative data represent mean ± SE, with various letters indicating significant difference (*p* ≤ 0.05) according to the Duncan test, as shown in Supplementary Table 3.

### 3.8 Wheat growth and yield parameters

**FIGURE 7:**
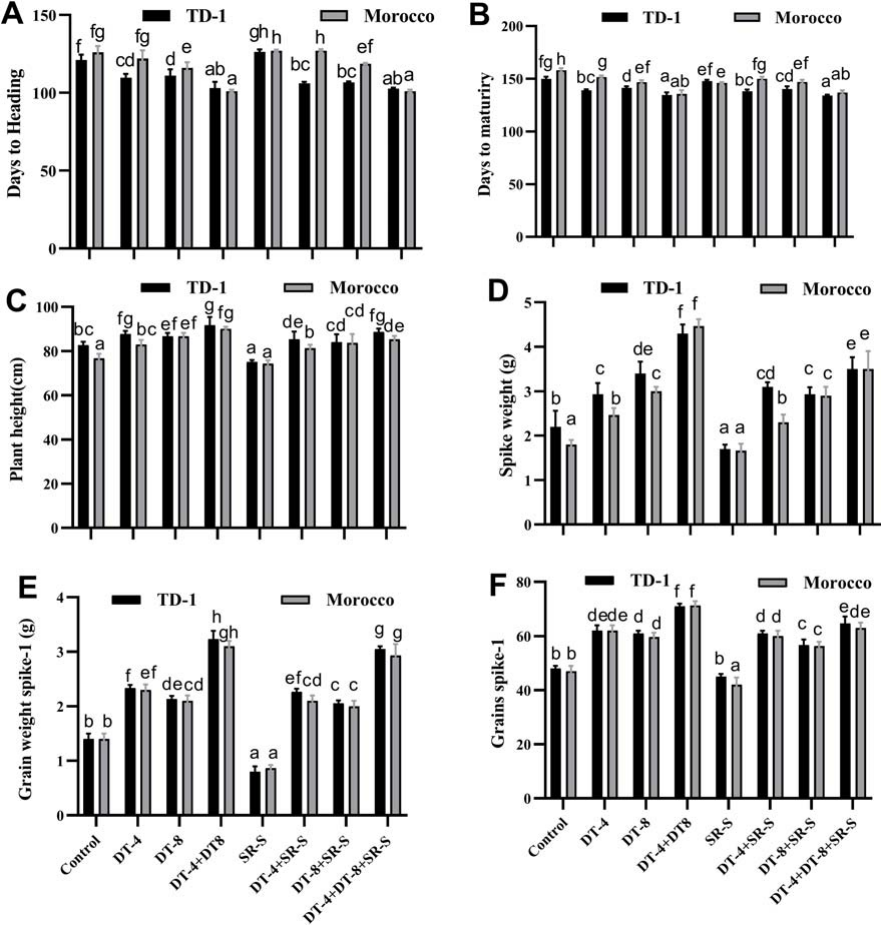
Growth and yield parameter in wheat: growth and yield were evaluated in 120 days old plants (30 dpi with stripe rust) pre-inoculated with DT-4 and DT-8 isolates for TD-1 and Morocco variety, **(A)** days to heading, **(B)** days to maturity, **(C)** plant height, **(D)** spike weight, **(E)** grain weight per spike, **(F)** grains per spike. Quantitative data are displayed as ± SE, with various letters indicating significant differences (*p* ≤ 0.05).

Treatments of DT-4 and DT-8 showed slight decreases in maturity and days to heading in comparison to the control, with DT-4+DT-8 demonstrating the shortest durations, indicating enhanced growth efficiency (Figures 7A,B). Plant height was significantly higher by DT-4+DT-8 compared to the control, while SR-S stress reduced it to around 75 cm in TD-1 and 74 cm in Morocco. The combination treatment DT-4+DT-8+SR-S restored height closer to 88 cm in TD-1 and 85 cm in Morocco, showing the efficiency of these isolates in enhancing growth under stress (Figure 7C). DT-4 and DT-8 enhanced spike weight significantly, reaching 4.3 g in TD-1 and 4.4 g in Morocco compared to the control (2.2 g and 1.8 g), with DT-4+DT-8+SR-S maintaining similar improvements under stress (Figure 7D). Grain yield per spike increased to 71 in DT-4+DT-8 treatments in both varieties, in comparison to the control (48 and 47). SR-S infection decreases the number of grains to 45 in TD-1 and 42 in Morocco, with combined treatments under SR-S showing partial restoration to 64 Grains spike-1 (Figure 7F). The grain weight per spike was highly affected by SR-S infection, decreasing from 1.4 to 0.8 g in TD-1, while DT-4+DT-8+SR-S showed improvement (3.05 and 2.9 g) under stress conditions (Figure 7E). Quantitative data represent mean ± SE, with various letters indicating significant difference (*p* ≤ 0.05) according to the Duncan test, as shown in Supplementary Table 4.

### 3.9 Effect of DT-4 and DT-8 on infected wheat leaves

**FIGURE 8:**
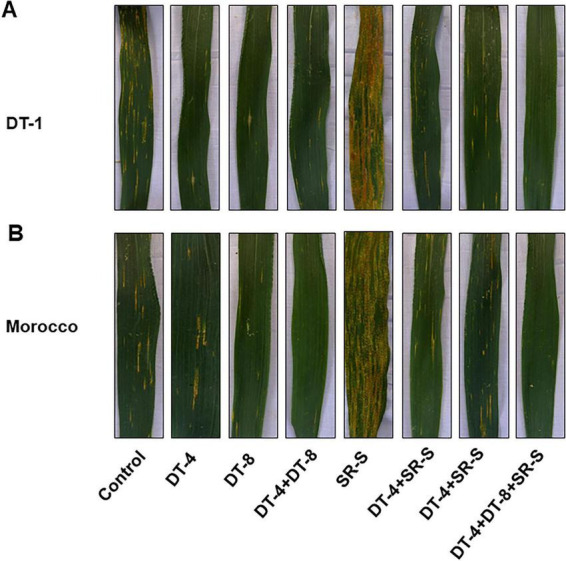
Disease scoring in wheat: disease scoring in wheat plants infected with SR-S and inoculated with DT-4 and DT-8 isolates quantified 10 days post-inoculation **(A)** TD-1 variety, **(B)** Morocco variety.

The wheat leaves infected by stripe rust were subjected to eight different treatments, including the application of biocontrol agents, DT-4 and DT-8, and their consortia. The visual observations of disease symptoms on the leaves of both varieties, TD-1 and Morocco, under different treatments are presented in Figure 8. The untreated wheat leaves exhibited extensive yellow rust infection (S reaction), with prominent chlorosis and necrosis, indicating severe disease progression. SR-S, used as a positive control, showed severe disease symptoms compared to the control and treated samples, indicating the severity of the infection. Individual treatment of leaves with DT-4 and DT-8 displayed less chlorosis and necrosis, suggesting partial inhibition of Pst. These results highlight the potential of DT-4 and DT-8 as biocontrol agents. Moreover, the combined application of DT-4 and DT-8 consortia exhibited the most potent suppression of disease symptoms compared to all other treatments (Figure 8).

### 3.10 Effect of DT-4 and DT-8 isolates on phytohormonal content of infected wheat plants

**FIGURE 9:**
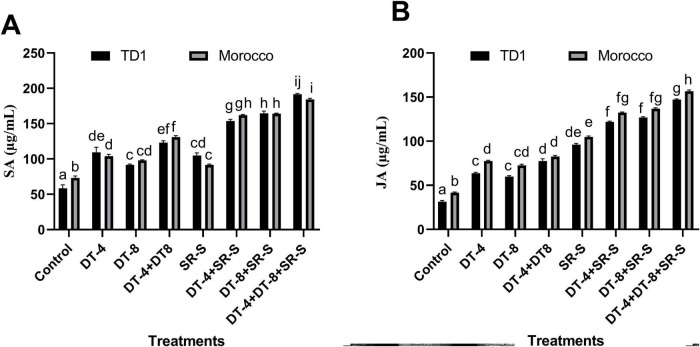
Jasmonic acid and salicylic acid quantification: effect of fungal isolates (DT-4 and DT-8) and pathogen SR-S co-inoculation on phytohormones in wheat **(A)** salicylic acid and **(B)** jasmonic acid content quantified 10 days post inoculation. Quantitative data are displayed as ± SE, with various letters indicating significant differences (*p* ≥ 0.05).

Salicylic acid, an important signaling molecule that helps the plant defense system, was determined in all eight treatments in both varieties. For TD1, a significant increase in SA content was recorded in DT-4 treatment (+87.44%), DT-8 treatment (+56.74%), and in consortia treatment (+110.81%) as compared to control plants (non-infected and non-inoculated). SA concentration was quantified, and results showed a significant increase in SR-S infected plants (+79.47%) as compared to the control. The DT-4, DT-8, and DT-4+DT-8 further raised the SA content to +161.84, +180.96, and +226.89%, respectively, as compared to the control. Similarly, in Morocco variety a progressive increase was noted in all treated as compared to the control. In DT-4, the value of SA rose to +41.78%, while DT-8 increased to +33.65%. The combined treatment DT-4+DT-8 showed a higher increase of +78.72%. In SR-S infected plants, an increase of +24.99% was recorded as compared to the control (non-infected and non-inoculated). A significant rise in DT-4, DT-8, and DT-4+DT-8 inoculated plants was observed with increasing +121.01, 123.76, and +151.56% as compared to the control (non-infected and non-inoculated), as shown in Figure 9A.

The results revealed a significant (*p* < 0.005) elevation in JA content of non-infected, endophyte-inoculated (DT-4 and DT-8) plants (+85.47% for DT-4 and +74.42% for DT-8) compared to control plants (non-infected and non-inoculated) of the TD-1 wheat variety. The combined inoculation of DT-4 and DT-8 further induced the accumulation of JA content by +125.69% in comparison to the control plants (non-infected and non-inoculated). JA content was quantified, and results showed that there was a significant increase in JA content +179.07% of SR-S plants without endophytic inoculation compared to control plants (non-infected and non-inoculated). Following the SR-S inoculation, JA content in endophyte-pre-inoculated plants with DT-4, DT-8, and DT-4 + DT-8 was further increased (+254.77, +269.15, and +327.02%, respectively) compared to the control seedlings (non-infected and non-inoculated). Similarly, in Morocco wheat variety a significant increase was noted in plant samples inoculated with DT-4 (+86.55%), DT-8 (+74.42%), and DT-4+DT-8 (+98.37%) as compared to the control (non-infected and non-inoculated). A significant increase was noted in JA content in plants infected with SR-S (+152.16%), while the DT-4, DT-8, and DT-4+DT-8 further evaluated the JA content (+218.22%, +229.43%, +275.13%) as compared to the control (non-infected and non-inoculated) (Figure 9B).

### 3.11 Effect of DT-4 and DT-8 isolates on *PR1* and *PR5* gene expression

**FIGURE 10:**
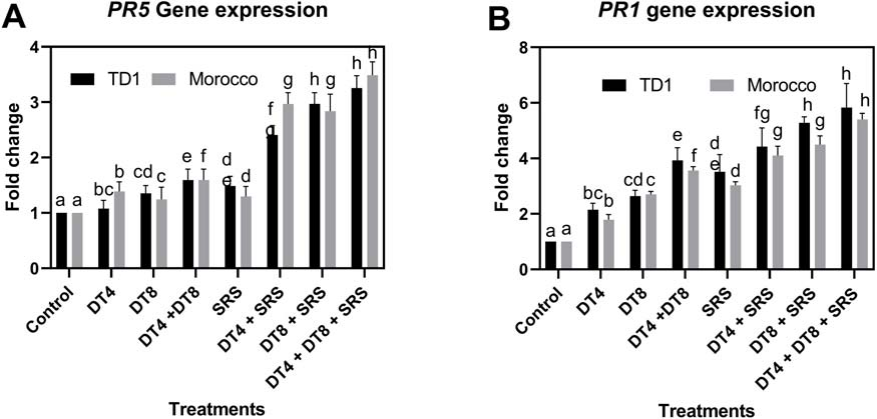
Quantitative real-time PCR (qRT-PCR) analysis of resistance genes. **(A)** Expression of PR5 gene. **(B)** Expression of PR1 gene in wheat leaves treated with *Curvularia lunata* and *Aspergillus fumigatus*, compared to the non-treated infected control. Data were normalized to the β-actin gene expression level. Quantitative data are displayed as ± SE, with various letters indicating significant differences (*p* ≥ 0.05).

The expression of defense-related genes like *PR1* and *PR5* was evaluated after 30 days of inoculation with Pst in both susceptible varieties, TD1 and Morocco. The expression level of the gene *PR5* was measured in all treatments of both varieties. Compared to the control, the *PR5* expression level increased 1.4-fold in TD1 after inoculation. For DT-4, DT-8, and the consortium, the SR-S infection boosted the *PR5* transcript level by 2.4, 2.9, and 3.2 times, respectively. Similarly, in Morocco, the expression was increased upon applying the pathogen and the endophytes. A 1.2-fold increase was recorded in SR-S infected compared to the control plants. Elevated expression values were recorded for DT-4 (2.9-fold) and DT-8 (2.8-fold) treatments with SR-S infection. A highest (3.4-fold) increase was recorded for the combined treatment of both endophytes with SR-S, as shown in Figure 10A. The expression of the *PR5* was significantly increased in all treatments, which reduced the disease severity. Similarly, the expression of the *PR1* gene was enhanced in SR-S infected wheat leaves treated with DT-4 and DT-8. The expression values in endophytes-treated plants were higher compared to those in untreated plants. In the TD1 variety, the expression was enhanced upon applying the endophytes to plants infected with SR-S. The highest fold change of 3.2 was recorded for DT-4 and DT-8 treated plants under infection. Similarly, the expression pattern of treatments was recorded in the same way, but with a different fold change. A high expression level (3.4-fold) was observed for endophytes consortium-treated plants under SR-S infection, as shown in Figure 10B.

## 4 Discussion

Stripe rust, caused by *Puccinia striiformis*, a worldwide primary infection of wheat, was previously primarily measured as a disease of cooler climates. However, recent waves of the disease have endangered this assumption, as new strains have developed increased variation to higher temperatures and regions near the equator (Waqar et al., 2018). The problem can be mitigated by knowledge about the infection, detecting resistance strains, and subsequently escalating in resistant varieties to reduce the infection cycle (Jamil et al., 2020). As biofertilizers, the endophytes can be a recovery method to expand soil microbial activity that encourages the expected soil microbiota, therefore provoking nutrient availability and decomposition of organic material (Fasusi et al., 2021).

Two native endophytic fungal strains separated from the roots of the *Phoenix dactylifera* L. plant investigated in the current study for their promising role were used as biological control agents against stripe rust in wheat plants. The wheat leaves infected with stripe rust were subjected to biocontrol agents (*Curvularia lunata* (DT-4) and *Aspergillus fumigatus* (DT-8), and their consortia. The roots of the date palm tree were utilized to isolate endophytic fungi. The isolated strains, their potential to alleviate biotic stress, were evaluated on wheat plants and assessed for plant growth-promoting traits. The strain isolated from the septic wheat leaves showed the symptoms of yellow rust revealed by hyphae, conidiophores, and conidia. The isolates assessed for their antifungal activities against yellow rust, had revealed significant antifungal activity against the invading pathogen and showed a high inhibitory effect by DT-4, while a moderate inhibitory effect was revealed by DT-8. The infected wheat leaves displayed characteristic yellow streaks, confirming the presence of stripe rust spores.

Previously, the plant growth-promoting and bio-control potential of *Aspergillus fumigatus* and *Curvularia lunata* has already been reported by many researchers. For example, *Aspergillus fumigatus* MCC1721, with special reference to indole-3-acetic acid production (Mahadik et al., 2024). *Curvularia lunata* AR11 and biochar stimulate synthetic silicon and potassium phosphate use efficiency, mitigate salt and drought stresses in Rice, and plant growth-promoting endophytic fungi *Asprgillus fumigatus* TS1 and *Fusarium proliferatum* BRL1 produce gibberellins and regulate plant endogenous hormones. The current research exposed and characterized the *Aspergillus fumigatus* and *Curvularia lunata* isolates for their growth-promoting as well as bio-control potential, which is indeed a novel property of these isolates. Moreover, these strains have produced growth-promoting metabolites such as IAA and GA, which proves that these strains have growth-promoting potential.

The endophytes, including *Aspergillus* species, elevate the level of plant growth-enhancing hormones auxin and gibberellins, as well as produce antifungal metabolites and enzymes, rendering them suitable as biocontrol agents (Chaudhary et al., 2022). Several endophytes, including *T. asperellum, T. afroharzianum, T. longibrachiatum, T. virens*, and *T. lixii*, are reported to enhance the photosynthetic potential of plants (Zeng et al., 2024). In the present study, the production of phytohormones gibberellic acid (GA) and indole-3-acetic acid (IAA 45 μg/mL) showed similar levels in the two isolates. Many other reports supported this outcome as there also maximum amount of IAA and GA was recorded (Attia et al., 2022). The production of metabolites such as flavonoids, phenols, and proline, were also similar in DT-4 and DT-8. Endophytes exhibit antagonistic effects against infection-causing phytopathogens and produce a range of bioactive antimicrobial compounds, along with different antioxidants to counteract infections. These observations support the findings reported previously by Gouda et al. (2023).

Endophytes, as potential agents, impart advantageous traits to the host plant and are well-established inoculants that enhance plant development directly or indirectly. Direct growth occurs by regulating the necessary nutrients, such as P and N, to control the levels of hormones, while indirectly through increased plant defense. The cytokinin, gibberellin, and indole acetic acid greatly assist plant growth (Chaudhary et al., 2022). The germination assay revealed a considerably positive impact of fungal isolates on wheat seeds. Tested seeds had shown enhanced germination rates, with visible seedling vigor, shoot development, and increased fresh weight. DT-4 showed a significant increase in fresh weight (3.5 g for Morocco), followed by DT-8 (3.0 g for Morocco), then control (1.5 g for Morocco). The highest shoot length was recorded in the DT-4 tested set (7 cm for Morocco), followed by DT-8 (6.5 cm for Morocco), and then in the control set (4 cm for Morocco). Thus, endophyte-pathogen interaction indirectly improved the overall growth of wheat plants by increasing plant defense, which is aligned with the observations reported previously by Chaudhary et al. (2022). The ability to colonize the plant tissue makes them a better biological agent than others in restoring natural compatibility once inoculated in plants (Rabiey et al., 2019). Inoculation by phyto-promotional fungal root endophyte *P. indica* boosts plant development, quick flowering, better seed production, and adjustments to stresses in different host plants such as *Triticum aestivum*, *Phaseolus vulgaris*, and *Cicer arientum* (Ansari et al., 2020). Root colonization analysis revealed substantial fungal colonization in both fungal treatments, indicating their role in promoting plant growth and mitigating pathogen-induced stress. It is also observed previously that the endophytes treatment considerably boosted key growth-promoting traits in rice plants, i.e., protein contents, chlorophyll, shoot and root length, dry biomass (Parveen et al., 2023). Pathogens produce various detrimental diseases in plants, decrease the photosynthetic rate, harm plant tissues, and result in stunted growth (Pérez-Bueno et al., 2019).

Metabolites generated by plants are crucial for their responses to various biotic and abiotic stressors. When a plant is under stress, these metabolites work as a signal or defensive compound to help the plant deal with these stresses (Mashabela et al., 2022). Several metabolites, such as phenolics and alkaloids, help significantly in disease resistance in plants. As quickly as the host plant is infected by the pathogen, it shows the production of phenolics that cause an escalation in host digestion (Wink, 2018). Similar findings were observed in our study as phenolic content noticeably increased upon pathogen infection in comparison to non-infected samples, thus reducing infection severity.

Enzymatic tools such as catalase, glutathione reductase, and superoxide dismutase, and non-enzymatic antioxidants, for instance, glutathione, ascorbic acid, flavonoids, and carotenoids, help in reducing oxidative stress by scavenging reactive oxygen species (ROS) (Das and Roychoudhury, 2014). POD and superoxide dismutase (SOD) are the main antioxidant enzymes, which aid in lipid peroxidation and defense against oxidative stress through pathogen attack (Hussain et al., 2017). The activity of the antioxidant enzymes ascorbic acid peroxidase, POD, SOD, and catalase was significantly increased in infected plants than in controls, associated with a decline in lipid peroxidation in the tested groups. Current outcomes also exposed that the inoculation of DT-4 and DT-8 endophytic fungi was advantageous to improve the POD and CAT activities and reduce ROS production. These findings demonstrated that the inoculation of DT-4 and DT-8 endophytic fungi could successfully lessen the harm caused by strip rust to leaves, promptly remove reactive oxygen species within a certain range, maintain the function of leaves, and thereby improve the growth and yield of wheat under rust stress. Similar findings were also reported previously by Hussain et al. (2017).

Resistance mechanisms by endophytes are supported via the stimulation of various defense compounds and enzymes at the site of the pathogen dose. POD and SOD are vital antioxidant enzymes that aid in resistance to oxidative stress and lipid peroxidation throughout pathogen stress (Birben et al., 2012). The activity of H_2_O_2_ increased in the plant under pathogen stress, compared to the control. An essential indicator, DAB staining, revealed the synthesis and buildup of H_2_O_2_. Our study demonstrated that after the SR-S condition, the CAT and POD activities were reduced, while DAB staining (H_2_O_2_ production) increased, indicating the harmful influence of strip rust on antioxidant enzyme systems, resulting in an imbalance of ROS and membrane deterioration, and hastening the aging of leaves. Crops’ ability to reduce oxidative damage brought on by pathogen stress can be improved by increasing the antioxidant enzyme activities (Rauf et al., 2022). This consistency of results assures that the validity of our results is in line with the findings reported previously (Birben et al., 2012).

MDA content under stress was elevated (3.28 Units/mg proteins for TD-1 and 3.05 Units/mg proteins for Morocco), compared to the control (1.72 Units/mg protein for TD-1 and 1.66 Units/mg for Morocco). The consortia reduced MDA levels (1.12 Units/mg proteins for TD-1 and 1.02 Units/mg proteins for Morocco), while double inoculation under stress further declined the level (1.90 Units/mg proteins for TD-1 and 2.05 Units/mg proteins for Morocco), indicating the stress-reducing role of the endophytic strains. The DAB staining revealed the increased H_2_O_2_ accumulation under pathogen stress, verified by the dark streaks in leaves. The control treatment showed a minimal streak. Application of DT-4 and DT-8 reduced streak intensity compared to pathogen stress. The consortia under stress showed the minimum streaks, indicative of reduced oxidative stress. Our observations are thus aligned with the statements reported previously by Birben et al. (2012) and Das and Roychoudhury (2014).

The SA-induced elicitation mechanism for pathogen resistance is essential for the expression of *PR* genes as well as for controlling the synthesis of defense-related substances like lignin, callose, and defense enzymes that are connected to both local and systemic acquired resistance. Defense-related genes play a crucial role in stripe rust resistance. Evaluations were conducted on the changes in the *PR* gene expression (*PR1, PR5*) and other important defensive enzymes involved in the DT-4 and DT-8 induced ISR and SAR in wheat. In all treatments, the high expression of *PR* protein genes (PR-1 and PR-5) was determined. The previous report also showed that endophytic *T. hamatum* elicited systemic immunity against mildew disease of pearl millet through the induction of defense proteins (*PR5, PR1*, hydroxyproline-rich glycoproteins; HRGPs) and defense enzymes (peroxidase, phenylalanine ammonia-lyase, –1,3-glucanase, and polyphenol oxidase (PPO) (Siddaiah et al., 2017). Moreover, pathogenesis-related proteins, including *PR-5*, *PR-1*, and –1,3- glucanase (*PR-2*), are often used as marker genes for the SA-dependent SAR, and basic chitinase (PR-3), plant-defense 1.2 gene (PDF1.2), and pathogenesis-related 4 (*PR-4*) are used as marker genes of the JA-dependent ISR (Jiang et al., 2018). Similarly, in several plants, PR-1 proteins have been used as markers for improved defense provided by pathogen-induced systemic acquired resistance (SAR) reported that PR1 is not only induced by SA but also recognized to be induced by a combination of ethylene and MeJA (precursor of JA) (Aboulila, 2022). In another independent study (Sakamoto et al., 2019), compared the effects of brassinosteroid or jasmonic acid treatment on the expression levels of *PR* genes. In the wild type, the expression levels of *PR1a, PR1b*, and *PR5* were significantly suppressed by brassinosteroid treatment and were significantly induced by jasmonic acid treatment. A study also revealed that *Aspergillus terreus* promotes the growth of tomato plants and induces resistance against *Pseudomonas syringae* pv. *tomato* by upregulation of the SAR-marker *PR1* in tomato plants (Rashad et al., 2022). *A. fumigatus* ranked the most effective in controlling the rice sheath blight fungal agent in the dual culture (Safari Motlagh et al., 2022). *PR1* plays a central role in regulating many of the responses to both SA and JA, notably by activating transcription of a battery of genes in response to rising SA levels and by modulating JA responses via the COI1-dependent pathway (Love et al., 2012). We therefore decided to carry out a time course analysis of the expression of the SA-responsive *PR* genes (*PR-1* and *PR-5*) in leaves of infected and non-infected wheat leaves. *PR-1* proteins have been used as markers in several plants for improved defense provided by pathogen-induced systemic acquired resistance (Salman et al., 2021). The most efficient way to induce a resistance response in wheat plants infected with stripe rust is to stimulate the *PR* protein gene expression. Therefore, in-depth molecular mechanisms involving potential genes, proteins, and metabolites associated with Pst resistance must be explored to enhance the disease resistance of wheat.

Seed-borne endophytic microorganisms can produce antimicrobial compounds, enzymes, and phytohormones, and improve plant growth and development (Shahzad et al., 2018, Ripa et al., 2019). The isolates proved to be capable of improving growth under pathogen stress. A slight decrease in days to heading and maturity compared to control was shown by both isolates, while consortia indicated the shortest durations, indicative of enhanced growth efficiency. The pathogen stress declined the wheat plant height (75 cm for TD-1 and 74 cm for Morocco) but was restored significantly by the consortia inoculation compared to the control (88 cm for TD-1 and 85 cm for Morocco). Similarly, the number of grains was reduced during pathogen stress (45 for TD-1 and 42 for Morocco), while consortia under stress revealed partial restoration (64 grains spike^–1^). The spike weight was enhanced significantly by both treatments (4.3 g for TD-1 and 4.4 g for Morocco) compared to the control (2.2 and 1.8 g). The grain yield per spike increased (71 in DT-4+DT-8) in both varieties, compared to the control (48 and 47). The grain weight per spike was highly decreased with stress (1.4–0.8 g for TD-1) but improved (3.05 and 2.9 g) by consortia treatment under stress. Hence, our findings support the previously reported results (Ripa et al., 2019; Shahzad et al., 2018).

## 5 Conclusion

Stripe rust is a warning threat to wheat farming in Pakistan. Owing to the precise land, statistics on the dispersion of rust are deficient. Sound-resourced conservatories need to be built to test the disease at the seedling stage to control its spread throughout the field. Resistance genes need to be recognized in the land-living races and are meant to be integrated into current cultivars. Constant monitoring, comprehensive epidemiological trials, and widespread pathological inquiry of rust samples in nearby association with adjacent countries to abide by an active management policy will be needed.

This study highlights the potential of indigenous fungal endophytes, *Curvularia lunata* (DT-4) and *Aspergillus fumigatus* (DT-8), as effective biocontrol agents against wheat stripe rust (*Puccinia striiformis*). Plants treated with biocontrol agents or their integration with stripe rust exhibited improved health and reduced pathogen impact compared to stress alone. The utilization of selected fungal endophytes DT-4 and DT-8 as possible bio-control agents is one of the quickest ways against stripe rust. The bio-control agents are efficient tools for relieving rust diseases and increasing resistance, thus mitigating biotic stress in wheat. These findings contribute to the growing knowledge of using fungal endophytes in integrated pest management strategies, offering an environmentally friendly and sustainable approach to combat stripe rust and improve wheat productivity. In summary, this study provides evidence that upon infection of Pst pathogen, DT-4 and DT-8 pre and post-inoculation enhance tolerance to the pathogen through boosted production of anti-pathogenic metabolites, anti-oxidant enzymes, defense-related proteins, induce SA, JA biosynthesis, and resistance genes (*PR*). Together, these responses enhance the overall defensive capacity of wheat against stripe rust, offering a viable alternative to synthetic fungicides for managing wheat yellow rust diseases.

## Data Availability

The raw data supporting the conclusions of this article will be made available by the authors, without undue reservation.

## References

[B1] Abbasi B. H. Ali J. Ali M. Zia M. Bokhari S. A. Khan M. A. (2016). Free radical scavenging activity in in vitro-derived tissues of Eruca sativa. *Toxicol. Ind. Health* 32 98–105. 10.1177/0748233713498450 24021434

[B2] Abd El-Rahman A. A. El-Shafei S. M. Ivanova E. V. Fattakhova A. N. Pankova A. V. El-Shafei M. A. (2014). Cytotoxicity of *Trichoderma* spp. cultural filtrate against human cervical and breast cancer cell lines. *Asian Pacific J. Cancer Prevent.* 15 7229–7234. 10.7314/apjcp.2014.15.17.7229 25227819

[B3] Aboulila A. A. (2022). Efficiency of plant growth regulators as inducers for improve systemic acquired resistance against stripe rust disease caused by *Puccinia striiformis* f. sp. tritici in wheat through up-regulation of PR-1 and PR-4 gene expression. *Physiol. Mol. Plant Pathol.* 121:101882. 10.1016/j.pmpp.2022.101882

[B4] Ali R. Gul H. Hamayun M. Rauf M. Iqbal A. Hussain A. (2022). Endophytic fungi controls the physicochemical status of maize crop under salt stress. *Pol. J. Environ. Stud.* 31 561–573. 10.15244/pjoes/134540

[B5] Ali S. Gladieux P. Leconte M. Gautier A. Justesen A. F. Hovmøller M. S. (2014a). Origin, migration routes and worldwide population genetic structure of the wheat yellow rust pathogen *Puccinia striiformis* f. sp. *tritici*. *PLoS Path.* 10:e1003903. 10.1371/journal.ppat.1003903 24465211 PMC3900651

[B6] Ali S. Gladieux P. Rahman H. Saqib M. S. Fiaz M. Ahmad H. (2014b). Inferring the contribution of sexual reproduction, migration and off-season survival to the temporal maintenance of microbial populations: A case study on the wheat fungal pathogen *Puccinia striiformis* f.sp. tritici. *Mol. Ecol.* 23 603–617. 10.1111/mec.12629 24354737

[B7] Amako K. Chen G.-X. Asada K. (1994). Separate assays specific for ascorbate peroxidase and guaiacol peroxidase and for the chloroplastic and cytosolic isozymes of ascorbate peroxidase in plants. *Plant Cell Physiol.* 35 497–504. 10.1093/oxfordjournals.pcp.a078621

[B8] Ansari W. A. Krishna R. Zeyad M. T. Singh S. Yadav A. (2020). “Endophytic actinomycetes-mediated modulation of defense and systemic resistance confers host plant fitness under biotic stress conditions,” in *Microbial versatility in varied environments*, eds Singh R. Manchanda G. Maurya I. Wei Y. (Singapore: Springer).

[B9] Arnon D. I. Whatley F. J. S. (1949). Is chloride a coenzyme of photosynthesis? *Science* 110 554–556. 10.1126/science.110.2865.554 15395399

[B10] Ashraf R. (2024). Determination, characterisation and combination of novel resistance genes to stripe and stem rust in wheat. *Acta Univ. Agric. Suec*. 19. 10.54612/a.4bqmv59tin

[B11] Attia H. Alamer K. Algethami B. Zorrig W. Hessini K. Gupta K. (2022). Gibberellic acid interacts with salt stress on germination, growth and polyamine gene expression in fennel (*Foeniculum vulgare* Mill.) seedlings. *Physiol. Mol. Biol. Plants* 28 607–622. 10.1007/s12298-022-01140-4 35465200 PMC8986931

[B12] Bates L. S. Waldren R. Teare I. J. (1973). Rapid determination of free proline for water-stress studies. *Plant Soil* 39 205–207. 10.1007/BF00018060

[B13] Birben E. Sahiner U. M. Sackesen C. Erzurum S. Kalayci O. (2012). Oxidative stress and antioxidant defense. *World Allergy Organ J.* 5 9–19. 10.1097/WOX.0b013e3182439613 23268465 PMC3488923

[B14] Brown J. K. Hovmøller M. S. (2002). Aerial dispersal of pathogens on the global and continental scales and its impact on plant disease. *Science* 297 537–541. 10.1126/science.1072678 12142520

[B15] Caverzan A. Casassola A. Brammer S. P. (2016). Antioxidant responses of wheat plants under stress. *Genet Mol Biol.* 39 1–6. 10.1590/1678-4685-GMB-2015-0109 27007891 PMC4807390

[B16] Chaudhary P. Agri U. Chaudhary A. Kumar A. Kumar G. (2022). Endophytes and their potential in biotic stress management and crop production. *Front. Microbiol.* 13:933017. 10.3389/fmicb.2022.933017 36325026 PMC9618965

[B17] Chugunkova T. Pastukhova N. Pirko Y. V. Blume Y. B. (2025). Genetic basis of resistance to wheat yellow rust. *Cytol. Genet.* 59 186–196. 10.3103/S0095452725020033

[B18] Conner R. Kuzyk A. (1988). Effectiveness of fungicides in controlling stripe rust, leaf rust, and black point in soft white spring wheat. *Can. J. Plant Pathol.* 10 321–326. 10.1080/07060668809501706

[B19] Conrath U. (2011). Molecular aspects of defence priming. *Trends Plant Sci.* 16 524–531. 10.1016/j.tplants.2011.06.004 21782492

[B20] Das K. Roychoudhury A. (2014). Reactive oxygen species (ROS) and response of antioxidants as ROS-scavengers during environmental stress in plants. *Front. Environ. Sci.* 2:53. 10.3389/fenvs.2014.00053

[B21] Dean R. Van Kan J. A. Pretorius Z. A. Hammond-Kosack K. E. Di Pietro A. Spanu P. D. (2012). The Top 10 fungal pathogens in molecular plant pathology. *Mol. Plant Pathol.* 13 414–430. 10.1111/j.1364-3703.2011.00783.x 22471698 PMC6638784

[B22] Din I. U. Khan S. Khan F. U. Khan M. Khan M. N. Hafeez A. (2023). Genetic characterization of advance bread wheat lines for yield and stripe rust resistance. *ACS Omega* 8 25988–25998. 10.1021/acsomega.3c01981 37521679 PMC10372943

[B23] Duan Z. Lv G. Shen C. Li Q. Qin Z. Niu J. (2014). The role of jasmonic acid signalling in wheat (*Triticum aestivum* L.) powdery mildew resistance reaction. *Eur. J. Plant Pathol.* 140 169–183. 10.1007/s10658-014-0453-2

[B24] El-Sharkawy H. H. Rashad Y. M. Ibrahim S. A. (2018). Biocontrol of stem rust disease of wheat using arbuscular mycorrhizal fungi and *Trichoderma* spp. *Physiol. Mol. Plant Pathol.* 103 84–91. 10.1016/j.pmpp.2018.05.002

[B25] Esmail S. M. Draz I. S. Saleem M. H. Mumtaz S. Elsharkawy M. M. (2022). *Penicillium simplicissimum* and *Trichoderma asperellum* counteract the challenge of *Puccinia striiformis* f. sp. tritici in wheat plants. *Egypt. J. Biol. Pest Control* 32:116. 10.1186/s41938-022-00614-7

[B26] Fasusi O. A. Cruz C. Babalola O. O. (2021). Agricultural sustainability: Microbial biofertilizers in rhizosphere management. *Agriculture* 11:163. 10.3390/agriculture11020163

[B27] Fernando K. Reddy P. Spangenberg G. C. Rochfort S. J. Guthridge K. M. (2021). Metabolic potential of Epichloë endophytes for host grass fungal disease resistance. *Microorganisms* 10:64. 10.3390/microorganisms10010064 35056512 PMC8781568

[B28] George N. M. Hany-Ali G. Abdelhaliem E. Abdel-Haleem M. (2024). Alleviating the drought stress and improving the plant resistance properties of *Triticum aestivum* via biopriming with *Aspergillus fumigatus*. *BMC Plant Biol.* 24:150. 10.1186/s12870-024-04840-z 38418956 PMC10900732

[B29] Gomes E. V. Ulhoa C. J. Cardoza R. E. Silva R. N. Gutiérrez S. (2017). Involvement of *Trichoderma harzianum* Epl-1 protein in the regulation of Botrytis virulence-and tomato defense-related genes. *Front. Plant Sci.* 8:880. 10.3389/fpls.2017.00880 28611802 PMC5446994

[B30] Gorin N. Heidema F. T. (1976). Peroxidase activity in golden delicious apples as a possible parameter of ripening and senescence. *J. Agricultural Food Chem.* 24 200–201. 10.1021/jf60203a043 1245669

[B31] Gouda M. Nassarawa S. S. Gupta S. D. Sanusi N. I. Nasiru M. M. (2023). Evaluation of carbon dioxide elevation on phenolic compounds and antioxidant activity of red onion (*Allium cepa* L.) during postharvest storage. *Plant Physiol. Biochem.* 200:107752. 10.1016/j.plaphy.2023.107752 37224628

[B32] Hayıt T. Erbay H. Varçın F. Hayıt F. Akci N. (2021). The classification of wheat yellow rust disease based on a combination of textural and deep features. *Multimed. Tools Appl.* 10.1007/s11042-023-15199-y [Epub ahead of print]. 37362723 PMC10173929

[B33] Horchani F. Bouallegue A. Bouazzi A. Abbes Z. (2025). Alleviating salt-Induced effects in tomato via simultaneous application of salicylic acid and potassium. *Russ. J. Plant Physiol.* 72:8. 10.1134/S1021443724609261

[B34] Hovmøller M. Justesen A. Brown J. (2002). Clonality and long-distance migration of *Puccinia striiformis* f. sp. tritici in north-west Europe. *Plant Pathol.* 51 24–32. 10.1046/j.1365-3059.2002.00652.x

[B35] Hussain I. Irshad M. Hussain A. Qadir M. Mehmood A. Urrehman M. (2025). Enhancing phosphorus uptake and mitigating lead stress in maize using the rhizospheric fungus *Talaromyces purpureogenus* PH7. *CLEAN–Soil Air Water* 53:e70006. 10.1002/clen.70006

[B36] Hussain I. Siddique A. Ashraf M. A. Rasheed R. Ibrahim M. Iqbal M. (2017). Does exogenous application of ascorbic acid modulate growth, photosynthetic pigments and oxidative defense in okra (*Abelmoschus esculentus* (L.) Moench) under lead stress? *Acta Physiol. Plant.* 39:144. 10.1007/s11738-017-2439-0

[B37] Isgandarova T. Y. Rustamova S. M. Aliyeva D. R. Rzayev F. H. Gasimov E. K. Huseynova I. M. (2024). Antioxidant and ultrastructural alterations in wheat during drought-induced leaf senescence. *Agronomy* 14:2924. 10.3390/agronomy14122924

[B38] Jamil S. Shahzad R. Ahmad S. Fatima R. Zahid R. Anwar M. (2020). Role of genetics, genomics, and breeding approaches to combat stripe rust of wheat. *Front. Nutrit.* 7:580715. 10.3389/fnut.2020.580715 33123549 PMC7573350

[B39] Jasrotia P. Kashyap P. L. Bhardwaj A. K. Kumar S. Singh G. P. (2018). Scope and applications of nanotechnology for wheat production: A review of recent advances. *Wheat Barley Res.* 10 1–14. 10.25174/2249-4065/2018/76672

[B40] Jiang H. Y. Zhang J. L. Yang J. W. Ma H. L. (2018). Transcript profiling and gene identification involved in the ethylene signal transduction pathways of creeping bentgrass (*Agrostis stolonifera*) during ISR response induced by butanediol. *Molecules* 23:706. 10.3390/molecules23030706 29558428 PMC6017539

[B41] Keutgen A. J. Pawelzik E. (2007). Modifications of strawberry fruit antioxidant pools and fruit quality under NaCl stress. *J. Agricultural Food Chem.* 55 4066–4072. 10.1021/jf070010k 17429984

[B42] Khalafallah A. A. Abo-Ghalia H. H. (2008). Effect of arbuscular mycorrhizal fungi on the metabolic products and activity of antioxidant system in wheat plants subjected to short-term water stress, followed by recovery at different growth stages. *J. Appl. Sci. Res.* 4 559–569.

[B43] Khalifa E. Oraby W. Saleem M. Hussein L. (2024). Predicting stripe rust disease severity in wheat using meteorological data with environmental response modeling. *Menoufia J. Plant Protect.* 9 115–131. 10.21608/mjapam.2024.269626.1034

[B44] Khan M. Rolly N. K. Al Azzawi T. N. I. Imran M. Mun B.-G. Lee I.-J. (2021). Lead (Pb)-induced oxidative stress alters the morphological and physio-biochemical properties of rice (*Oryza sativa* L.). *Agronomy* 11:409. 10.3390/agronomy11030409

[B45] Khan R. A. A. Najeeb S. Hussain S. Xie B. Li Y. (2020). Bioactive secondary metabolites from *Trichoderma spp*. against phytopathogenic Fungi. *Microorganisms* 8:817. 10.3390/microorganisms8060817 32486107 PMC7356054

[B46] Khanfri S. Boulif M. Lahlali R. (2018). Yellow rust (*Puccinia striiformis*): a serious threat to wheat production worldwide. *Notulae Sci. Biol.* 10 410–423. 10.25835/nsb10310287 40824611

[B47] Kiani T. Mehboob F. Hyder M. Z. Zainy Z. Xu L. Huang L. (2021). Control of stripe rust of wheat using indigenous endophytic bacteria at seedling and adult plant stage. *Sci. Rep.* 11:14473. 10.1038/s41598-021-93939-6 34262108 PMC8280153

[B48] Kumar D. Yusuf M. A. Singh P. Sardar M. Sarin N. B. (2014). Modulation of antioxidant machinery in α-tocopherol-enriched transgenic *Brassica juncea* plants tolerant to abiotic stress conditions. *Protoplasma* 250 1079–1089. 10.1007/s00709-013-0484-0 23361901

[B49] Love A. J. Geri C. Laird J. Carr C. Yun B. W. Loake G. J. (2012). Cauliflower mosaic virus protein P6 inhibits signaling responses to salicylic acid and regulates innate immunity. *PLoS One* 7:e47535. 10.1371/journal.pone.0047535 23071821 PMC3469532

[B50] Maclachlan S. Zalik S. (1963). Plastid structure, chlorophyll concentration, and free amino acid composition of a chlorophyll mutant of barley. *Can. J. Botany* 41 1053–1062. 10.1139/b63-088

[B51] Mahadik S. P. Patil S. V. Kumudini B. S. (2024). Bioprospecting rhizosphere fungi endowed with multifarious plant growth-promoting potential to enhance finger millet growth under salinity stress. *Plant Growth Regul.* 104 1483–1505. 10.1007/s10725-024-01234-x

[B52] Mashabela M. D. Piater L. A. Steenkamp P. A. Dubery I. A. Tugizimana F. Mhlongo M. I. (2022). Comparative metabolite profiling of wheat cultivars (*Triticum aestivum*) reveals signatory markers for resistance and susceptibility to stripe rust and aluminium (Al3+) toxicity. *Metabolites* 12:98. 10.3390/metabo12020098 35208172 PMC8877665

[B53] Mehdi F. Wu Y. Gan Y. Cao Z. Jiang S. Zan L. (2025). Endophytic microbes enhance sugarcane defense against *Sporisorium scitamineum* by activating calcium signaling and stress-responsive traits. *Curr. Plant Biol.* 43:100506. 10.1016/j.cpb.2025.100506

[B54] Mohammadkhani N. Heidari R. (2008). Drought-induced accumulation of soluble sugars and proline in two maize varieties. *World Appl. Sci. J.* 3 448–453.

[B55] Pan H. Zheng X. Tian X. Geng Y. Zhang X. Xiao S. (2022). Toward sustainable crop production in China: A co-benefits evaluation. *J. Clean. Product.* 361:132285. 10.1016/j.jclepro.2022.132285

[B56] Parveen S. Mohiddin F. A. Bhat M. A. Baba Z. A. Jeelani F. Bhat M. A. (2023). Characterization of endophytic microorganisms of rice (*Oryza sativa* L.) potentials for blast disease biocontrol and plant growth promoting agents. *Phyton* 92 3021–3041. 10.32604/phyton.2023.030921

[B57] Pérez-Bueno M. L. Pineda M. Barón M. (2019). Phenotyping plant responses to biotic stress by chlorophyll fluorescence imaging. *Front. Plant Sci.* 10:1135. 10.3389/fpls.2019.01135 31620158 PMC6759674

[B58] Rabiey M. Hailey L. E. Roy S. R. Grenz K. Al-Zadjali M. A. Barrett G. A. (2019). Endophytes vs tree pathogens and pests: can they be used as biological control agents to improve tree health? *Eur. J. Plant Pathol.* 155 711–729. 10.1007/s10658-019-01814-y

[B59] Rahman K. U. Ali K. Rauf M. Arif M. (2023). *Aspergillus* nomiae and fumigatus ameliorating the hypoxic stress induced by waterlogging through ethylene metabolism in Zea mays L. *Microorganisms* 11:2025. 10.3390/microorganisms11082025 37630585 PMC10459883

[B60] Rashad Y. M. Abdalla S. A. Shehata A. S. (2022). *Aspergillus flavus* YRB2 from *Thymelaea hirsuta* (L.) Endl., a non-aflatoxigenic endophyte with ability to overexpress defense-related genes against Fusarium root rot of maize. *BMC Microbiol.* 22:229. 10.1186/s12866-022-02651-6 36175855 PMC9524039

[B61] Rauf M. Ur-Rahman A. Arif M. Gul H. Ud-Din A. Hamayun M. (2022). Immunomodulatory molecular mechanisms of *Luffa cylindrica* for downy mildews resistance induced by growth-promoting endophytic fungi. *J. Fungi* 8:689. 10.3390/jof8070689 35887445 PMC9324744

[B62] Ribeiro B. Valentão P. Baptista P. Seabra R. M. Andrade P. B. (2007). Phenolic compounds, organic acids profiles and antioxidative properties of beefsteak fungus (*Fistulina hepatica*). *Food Chem. Toxicol.* 45 1805–1813. 10.1016/j.fct.2007.03.015 17493733

[B63] Ripa F. A. Cao W. D. Tong S. Sun J. G. (2019). Assessment of plant growth promoting and abiotic stress tolerance properties of wheat endophytic fungi. *BioMed Res. Intern.* 2019:6105865. 10.1155/2019/6105865 31032353 PMC6457323

[B64] Saad A. Saad M. Ibrahim N. El-Hadedy D. Ibrahim E. El-Din A. (2019). Evaluation of *Aspergillus tamarii* NRC 3 biomass as a biosorbent for removal and recovery of heavy metals from contaminated aqueous solutions. *Bull. Natl. Res. Centre* 43:10. 10.1186/s42269-019-0046-5

[B65] Safari Motlagh M. R. Jahangiri B. Kulus D. Tymoszuk A. Kaviani B. (2022). Endophytic fungi as potential biocontrol agents against rhizoctonia solani J.G. Kühn, the causal agent of rice sheath blight disease. *Biology* 11:1282. 10.3390/biology11091282 36138761 PMC9495574

[B66] Sajid Ali S. A. Shah S. J. A. Khalil I. Raman H. Maqbool K. Waseem Ullah W. U. (2009). Partial resistance to yellow rust in introduced winter wheat germplasm in north of Pakistan. *Aus. J. Crop Sci.* 3 37–43.

[B67] Sakamoto T. Kitano H. Fujioka S. (2019). ERECT LEAF1 suppresses jasmonic acid response in rice by decreasing OsWRKY4 stability. *Plant Signal. Behav.* 14:1559578. 10.1080/15592324.2018.1559578 30572766 PMC6351086

[B68] Salman E. K. Badr E. S. Ghoniem K. E. Aboulila A. A. Emeran A. A. (2021). Role of silymarin induced rice immunity against blast pathogen *Magnaporthe oryzae* through regulation of resistance genes expression. *Physiol. Mol. Plant Pathol.* 115:101678. 10.1016/j.pmpp.2021.101678

[B69] Shafi S. Tahir M. Khan M. A. Bhat M. A. Kumar U. Kumar S. (2022). Trait phenotyping and SSR markers characterization of wheat (*Triticum aestivum* L.) germplasm for breeding early maturing wheat’s for Western-Himalayas. *Genet. Resour. Crop Evol*. 69, 755–770.

[B70] Shahzad R. Khan A. L. Bilal S. Asaf S. Lee I.-J. (2018). What is there in seeds? Vertically transmitted endophytic resources for sustainable improvement in plant growth. *Front. Plant Sci.* 9:24. 10.3389/fpls.2018.00024 29410675 PMC5787091

[B71] Siddaiah C. N. Satyanarayana N. R. Mudili V. Kumar Gupta V. Gurunathan S. Rangappa S. (2017). Elicitation of resistance and associated defense responses in *Trichoderma hamatum* induced protection against pearl millet downy mildew pathogen. *Sci. Rep.* 7:43991. 10.1038/srep43991 28322224 PMC5359564

[B72] Singh R. P. William H. M. Huerta-Espino J. Rosewarne G. (2004). “Wheat rust in Asia: Meeting the challenges with old and new technology “New directions for a diverse planet”,” in *Proceedings of the 4th International Crop Science Congress*, (Brisbane, QLD).

[B73] Song F.-L. Gan R.-Y. Zhang Y. Xiao Q. Kuang L. Li H.-B. J. (2010). Total phenolic contents and antioxidant capacities of selected Chinese medicinal plants. *Intern. J. Mol. Sci.* 11 2362–2372. 10.3390/ijms11062362 20640157 PMC2904921

[B74] Sørensen C. K. Thach T. Hovmøller M. S. (2016). Evaluation of spray and point inoculation methods for the phenotyping of *Puccinia striiformis* on wheat. *Plant Dis.* 100 1064–1070. 10.1094/PDIS-12-15-1477-RE 30682276

[B75] Upadhyaya S. Khatiwora E Saikia L. R. (2010). Comparison of total phenol and flavonoid content in *Adhatoda vasica* Nees.: Grown using different organic manure. *J. Pharmacy Res.* 3, 2408–2409.

[B76] Wang M. N. Chen X. (2013). First report of Oregon grape (*Mahonia aquifolium*) as an alternate host for the wheat stripe rust pathogen (*Puccinia striiformis* f. sp. *tritici*) under artificial inoculation. *Plant Dis.* 97 839–839. 10.1094/PDIS-09-12-0864-PDN 30722629

[B77] Waqar A. Khattak S. H. Begum S. Rehman T. Shehzad A. Ajmal W. (2018). Stripe rust: A review of the disease, Yr genes and its molecular markers. *Sarhad J. Agriculture* 34 188–201. 10.17582/journal.sja/2018/34.1.188.201

[B78] Wink M. (2018). Plant secondary metabolites modulate insect behavior-steps toward addiction? *Front. Physiol.* 9:364. 10.3389/fphys.2018.00364 29695974 PMC5904355

[B79] Wu J. Ma S. Niu J. Sun W. Dong H. Zheng S. (2025). Genomics-driven discovery of superior alleles and genes for yellow rust resistance in wheat. *Nat. Genet.* 57 2017–2027. 10.1038/s41588-025-02259-2 40696174

[B80] Zeng Q. Dong J. Lin X. Zhou X. Xu H. (2024). Isolation and identification of *Acer truncatum* endophytic fungus *Talaromyces verruculosus* and evaluation of its effects on insoluble phosphorus absorption capacity and growth of Cucumber seedlings. *J. Fungi* 10:136. 10.3390/jof10020136 38392808 PMC10890576

[B81] Zhang B. Zhao J. Huang J. Wang X. Guo Z. Jia Q. (2024). Aggressiveness of *Puccinia striiformis* f. sp. tritici Isolates at high temperatures: A study case in core oversummering area of gansu as inoculum source. *Plants* 13:3518. 10.3390/plants13243518 39771216 PMC11678112

[B82] Zhang Y. J. Zhang S. Liu X. Z. Wen H. A. Wang M. (2010). A simple method of genomic DNA extraction suitable for analysis of bulk fungal strains. *Lett. Appl. Microbiol.* 51 114–118. 10.1111/j.1472-765X.2010.02867.x 20536704

