# Supplementary material for: Integrated biocontrol strategies using indigenous fungal endophytes Aspergillus fumigatus and Curvularia lunata against wheat stripe rust
Source: Front Microbiol. 2025 Dec 19;16:1683295. doi: 10.3389/fmicb.2025.1683295 (PMC12757442; doi:10.3389/fmicb.2025.1683295)
Supplement: Supplementary file 1 [file Table_1.docx]

**
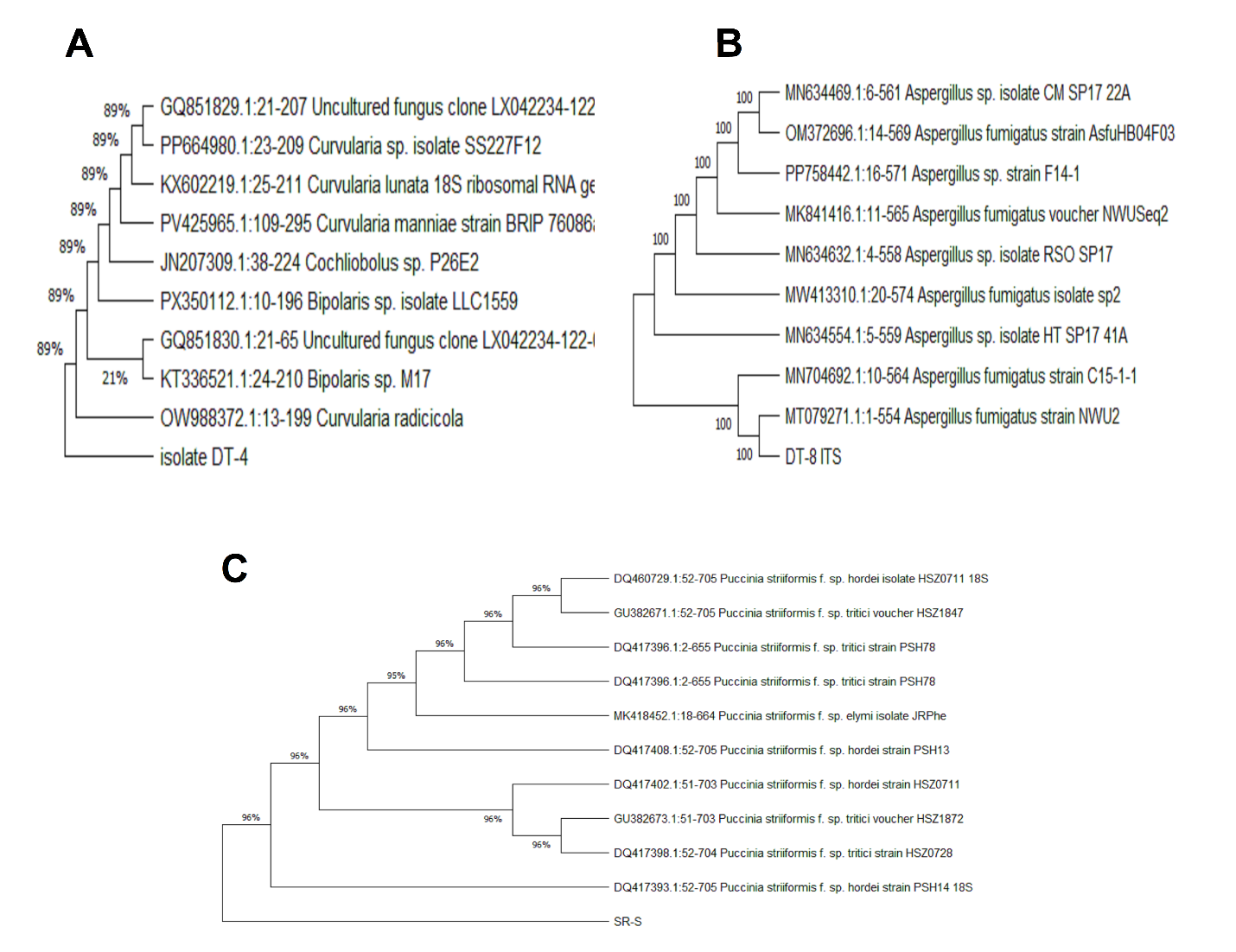
**

**Figure 1. Phylogenetic analysis of two fungus isolates DT4 and DT8 (A) DT4 identified as Curvularia lunata (B) DT-8 identifies as Aspergillus fumigatus (C) *Puccinia striiformis* (SR-S)**

**Figure 2. Wheat seedling length inoculated with DT-4 and DT-8 Isolates. Macroscopic images for the TD1 and Morocco variety of wheat.**

**Table 1.** Quantitative analysis of Fresh and Dry Biomass of fungal isolates and different metabolites. Quantitative data represent mean ± SE.

| Fungus Isolates | Biomass FW(mg/ml) | Biomass DW(mg/ml) | DPPH  (%) | Proline  (µg/ml) | Phenols (µg/ml) | Flavonoids (µg/ml) | IAA  (µg/ml) | GA  (µg/ml) |
| --- | --- | --- | --- | --- | --- | --- | --- | --- |
| DT-4 | 72.1±0.12 | 19.7±0.1 | 90.3±0.33 | 187.38±6.1 | 275.25±7.38 | 287.29±9.04 | 45.86±1.1 | 24.37±0.45 |
| DT-8 | 83.1±0.09 | 24.6±0.1 | 95.33±0.33 | 186.9±8.27 | 268.96±8.85 | 269.58±9.61 | 44.42±1.93 | 22.96±0.32 |

**Table 2.** Quantitative analysis of metabolites in eight different treatments of TD1 and Morocco wheat varieties. Quantitative data represent mean ± SE, with various letters indicated significant difference (p≤0.05) according to Duncan test.

| Treatments | Varieties | Flavonoids | Sugar | Phenols | Protein |
| --- | --- | --- | --- | --- | --- |
| Control | TD-1 | 1.33±0.029c | 23.35±0.22d | 8.5±0.24a | 25.35±0.22d |
|  | Morocco | 1.14±0.007b | 20.32±0.23c | 8.17±0.07a | 22.32±0.23c |
| DT-4 | TD-1 | 2.19±0.036f | 35.35±0.22k | 11.68±0.1f | 30.72±0.38fg |
|  | Morocco | 2.23±0.0615fg | 33.81±0.51j | 11.51±0.15f | 29.72±0.38f |
| DT-8 | TD-1 | 2.05±0.0399e | 28.6±0.29h | 11.45±0.22ef | 28.31±0.23e |
|  | Morocco | 1.84±0.039d | 27.17±0.08g | 11.5±0.13f | 28±0.51e |
| DT4+DT-8 | TD-1 | 2.43±0.019h | 39.6±0.29m | 11.34±0.26ef | 34.37±0.21h |
|  | Morocco | 2.23±0.022fg | 36.05±0.17k | 12.03±0.17f | 30.4±0.26fg |
| SRS | TD-1 | 0.92±0.006a | 16.31±0.04b | 7.66±0.56b | 18.31±0.04b |
|  | Morocco | 0.85±0.025a | 12.36±0.08a | 7.4±0.63ab | 15.37±0.08gh |
| SRS+DT-4 | TD-1 | 2.33±0.019g | 26.31±0.23f | 10.65±0.18de | 31.84±0.42f |
|  | Morocco | 2.25±0.02fg | 24.9±0.54e | 10.37±0.09cd | 29.9±0.54h |
| SRS+DT-8 | TD-1 | 2.29±0.03fg | 30.37±0.21i | 10.34±0.06cd | 32.4±0.47fg |
|  | Morocco | 2.31±0.03g | 28.72±0.38h | 9.62±0.13cd | 31±0.75h |
| SRS+DT4+DT8 | TD-1 | 2.46±0.035h | 39.15±0.08m | 11.42±0.11ef | 34±1.09g |
|  | Morocco | 2.26±0.033hg | 38.2±0.09l | 11.55±0.14f | 31.22±0.47fg |

**Table 3.** Quantitative analysis of antioxidants in eight different treatments of TD1 and Morocco wheat varieties. Quantitative data represent mean ± SE, with various letters indicated significant difference (p≤0.05) according to Duncan test.

| Treatments | Varieties | Catalase | APX | H202 | sod | MDA | POD |
| --- | --- | --- | --- | --- | --- | --- | --- |
| Control | TD-1 | 2.233±0.004a | 1.45±0.009a | 2.72±0.007e | 8.66±0.019a | 1.72±0.008g | 1.66±0.019a |
|  | Morocco | 2.59±0.015b | 1.41±0.038a | 2.66±0.016e | 8.39±0.054a | 1.66±0.016f | 1.39±0.055a |
| DT-4 | TD-1 | 3.79±0.03d | 2.54±0.056e | 2.53±0.002d | 16.42±0.11f | 1.53±0.002e | 3.65±0.18b |
|  | Morocco | 3.86±0.15d | 2.41±0.03d | 2.27±0.001c | 15.55±0.14e | 1.27±0.002c | 3.37±0.096d |
| DT-8 | TD-1 | 3.25±0.017c | 2.17±0.028c | 2.44±0.01d | 14.65±0.18d | 1.44±0.015d | 4.5±0.24cd |
|  | Morocco | 3.07±0.052c | 2.07±0.038c | 2.22±0.033c | 13.37±0.09c | 1.42±0.06d | 4.17±0.077cd |
| DT4+DT-8 | TD-1 | 4.73±0.021f | 3.85±0.069i | 1.34±0.015b | 20.5±0.24g | 1.13±0.0021b | 5.34±0.066e |
|  | Morocco | 4.35±0.01e | 3.58±0.003h | 1.22±0.138a | 20.17±0.076g | 1.02±0.003a | 4.62±0.13ed |
| SRS | TD-1 | 3.73±0.02d | 1.89±0.03b | 6.37±0.006k | 14.61±0.12d | 3.28±0.005m | 3.61±0.122b |
|  | Morocco | 3.35±0.013c | 1.78±0.03b | 6.02±0.004j | 14.67±0.19d | 3.05±0.01l | 3.671±0.19b |
| SRS+DT-4 | TD-1 | 5.55±0.1ij | 3.34±0.11g | 4.8±0.011i | 13.34±0.066c | 2.44±0.014k | 4.45±0.22cd |
|  | Morocco | 5.36±0.018hi | 3.14±0.02f | 4.65±0.018h | 12.62±0.13b | 2.47±0.003k | 4.5±0.13cd |
| SRS+DT-8 | TD-1 | 5.07±0.04gh | 3.26±0.024fg | 4.16±0.019g | 15.68±0.094e | 2.37±0.006j | 4.34±0.26cd |
|  | Morocco | 4.84±0.03fg | 3.23±0.012fg | 4.08±0.008g | 14.51±0.14d | 2.02±0.004i | 4.69±0.16d |
| SRS+DT4+DT8 | TD-1 | 5.72±0.007j | 4.15±0.0069j | 3.62±0.008f | 22.56±0.16h | 1.9±0.002h | 7.68±0.094f |
|  | Morocco | 5.7±0.33j | 4.13±0.013j | 3.55±0.015f | 23.35±0.2i | 2.05±0.018i | 7.51±0.14f |

**Table 4.** Analysis of Agronomic parameters in eight different treatments of TD1 and Morocco wheat varieties. Quantitative data represent mean ± SE, with various letters indicated significant difference (p≤0.05) according to Duncan test.

| Treatments | Varieties | Days to Heading | Days to Maturity | Height | Spike Weight | Grain.Spike^-1^ | Grain Weight |
| --- | --- | --- | --- | --- | --- | --- | --- |
| Control | TD-1 | 121±2f | 150±1.15fg | 82.66±0.88bc | 2.2±0.2b | 48±0.57b | 1.4±0.05b |
|  | Morocco | 126±2.3fg | 158±1.15h | 76.66±1.2a | 1.8±0.057a | 47±1.15b | 1.4±0.05b |
| DT-4 | TD-1 | 109.66±1.45cd | 139±0.57bcd | 87.66±0.8defg | 2.93±0.14c | 62±1.15defg | 2.33±0.03f |
|  | Morocco | 122±3.05fg | 151.66±0.88gh | 83±1.15bc | 2.46±0.088b | 62±1.15defg | 2.3±0.057ef |
| DT-8 | TD-1 | 111±2.3d | 141.33±0.88d | 86.66±0.88defg | 3.4±0.15defg | 61±0.57d | 2.13±0.03cde |
|  | Morocco | 116±2.08e | 146.66±1.2ef | 86.66±0.88defg | 3±0.057c | 59.66±0.88d | 2.1±0.056cd |
| DT4+DT-8 | TD-1 | 103±2.3ab | 134.66±1.45a | 91.66±2.18gh | 4.3±0.1f | 71±0.57f | 3.23±0.09h |
|  | Morocco | 101±0.57ab | 135.66±2ab | 90±0.57fg | 4.46±0.08f | 71.33±0.88f | 3.1±0.054gh |
| SRS | TD-1 | 126.33±0.88gh | 148±0.57ef | 75±0.57a | 1.7±0.05a | 45±0.57b | 0.8±0.058a |
|  | Morocco | 127.06±0.4h | 146±0.57e | 74.33±0.88a | 1.66±0.08a | 42±1.5a | 0.86±0.04a |
| SRS+DT-4 | TD-1 | 106±0.57bc | 138.33±0.88bcd | 85.33±2.02cde | 3.1±0.05cd | 61±0.57d | 2.26±0.03defg |
|  | Morocco | 127±0.57h | 150±1.15fg | 81.33±0.88b | 2.3±0.1b | 60±1.15d | 2.1±0.057cd |
| SRS+DT-8 | TD-1 | 106.66±0.33bcd | 140.33±1.45cd | 84±2.08cd | 2.93±0.088c | 56.66±1.2c | 2.05±0.029c |
|  | Morocco | 118.66±0.33ef | 147±1.15ef | 83.66±2.33cd | 2.9±0.11c | 56.33±0.8c | 2±0.057c |
| SRS+DT4+DT8 | TD-1 | 102.66±0.33ab | 134±0.57a | 88.66±0.88efg | 3.5±0.15e | 64.66±1.45e | 3.05±0.028g |
|  | Morocco | 101±0.57a | 137±1.15abc | 85.33±0.88cde | 3.5±0.23e | 63±1.15defg | 3±0.12g |
